# The ‘emodin family’ of fungal natural products–amalgamating a century of research with recent genomics-based advances

**DOI:** 10.1039/d2np00040g

**Published:** 2022-10-12

**Authors:** Kate M. J. de Mattos-Shipley, Thomas J. Simpson

**Affiliations:** a School of Biological Sciences, University of Bristol 24 Tyndall Avenue Bristol BS8 1TQ UK kate_dms@hotmail.com; b School of Chemistry, University of Bristol Cantock's Close Bristol BS8 1TS UK

## Abstract

Covering: up to 2022

A very large group of biosynthetically linked fungal secondary metabolites are formed *via* the key intermediate emodin and its corresponding anthrone. The group includes anthraquinones such as chrysophanol and cladofulvin, the grisandienes geodin and trypacidin, the diphenyl ether pestheic acid, benzophenones such as monodictyphenone and various xanthones including the prenylated shamixanthones, the agnestins and dimeric xanthones such as the ergochromes, cryptosporioptides and neosartorin. Such compounds exhibit a wide range of bioactivities and as such have been utilised in traditional medicine for centuries, as well as garnering more recent interest from the pharmaceutical sector. Additional interest comes from industries such as textiles and cosmetics due to their use as natural colourants. A variety of biosynthetic routes and mechanisms have been proposed for this family of compounds, being altered and updated as new biosynthetic methods develop and new results emerge. After nearly 100 years of such research, this review aims to provide a comprehensive overview of what is currently known about the biosynthesis of this important family, amalgamating the early chemical and biosynthetic studies with the more recent genetics-based advances and comparative bioinformatics.

## Abstract 2

A large and diverse family of fungal compounds are those related to the anthraquinone emodin. By viewing this family as a whole and combining our genetic and chemical understanding, we gain detailed insights into their biosynthetic origins.

## Graphical abstract

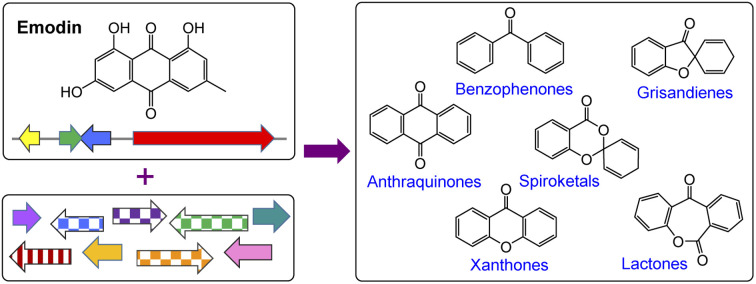

## 1 Introduction

**Fig. 1:**
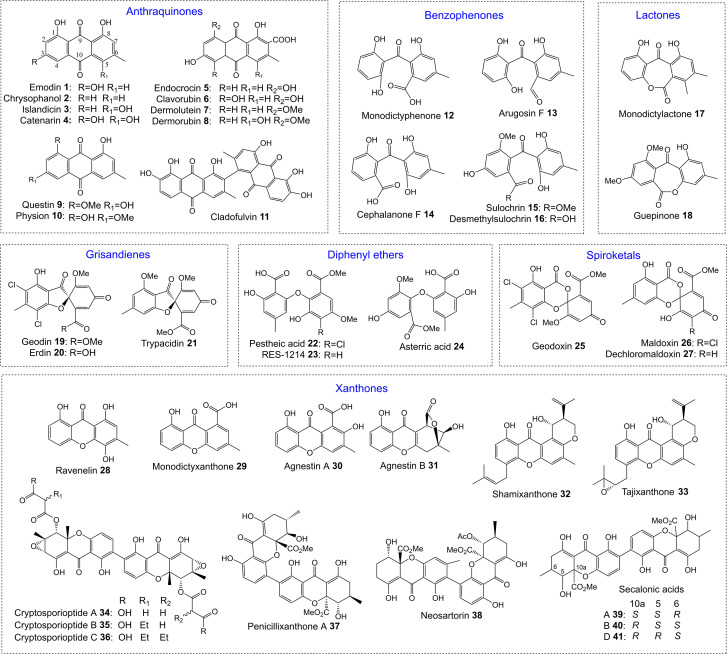
Examples of fungal natural products belonging to the emodin family of secondary metabolites.

One of the largest families of fungal natural products are those related to the anthraquinone emodin 1. Although biosynthetically linked, they are chemically diverse, including not only anthraquinones (*e.g.*1–11) but also benzophenones (*e.g.*12–16), lactones (*e.g.*17, 18), grisandienes (*e.g.*19–21), diphenyl ethers (*e.g.*22–24), spiroketals (*e.g.*25–27) and both monomeric (*e.g.*28–33) and dimeric (*e.g.*34–41) xanthones (Fig. 1). It is worth immediately clarifying that although the emodin-related family of natural products includes all of the above structural classes, not all compounds belonging to those classes also belong to the ‘emodin family’. The aflatoxins and related sterigmatocystins, for example, have isocoumarin and xanthone cores, and while their biosynthesis shares many of the same chemistries and enzyme classes as the compounds described here, they are not emodin derived, being formed *via* another anthraquinone, norsolorinic acid. Other structurally similar compounds such as such as griseofulvin 42 and the griseoxanthones are striking examples of convergent evolution, being structurally very similar to emodin-related grisandienes and xanthones such as geodin 19 and ravenelin 28 (see Fig. 1) but being biosynthesised *via* distinctly different routes (see Section 4.3.2; Box 2). Such compounds will only be discussed in this review in instances where theories regarding their biosynthetic pathways have directly aided – or in some cases hindered – understanding of the emodin family of compounds.

As a family of secondary metabolites, emodin 1 and related compounds have a long and rich history. Emodin 1 was one of the earliest fungal secondary metabolites to be identified and structurally characterised.^1,2^ It has been isolated from a range of filamentous fungi including both basidiomycetes and ascomycetes.^3^ In addition to fungi, emodin is produced by actinomycetes, plants and lichens, and is the major bioactive compound in plants such as *Rheum palmatum* and *Polygonum cuspidatum*, which have been used in traditional medicine for centuries. In fact, the name emodin is derived from the latin name for a medicinal plant called *Rheum emodi*, the Himalayan rhubarb. Used traditionally as a purgative agent, emodin 1 and related anthraquinones have been shown more recently to display a wide range of potentially applicable bioactivities, including antiviral, antifungal, antitumour and anti-inflammatory.^4^ Anthraquinones are also important as natural pigments and colourants, and are used in many industries such as textile dying and cosmetics.^5^

In the decades following the isolation of emodin 1 in 1924, many compounds now known to be biosynthetically related were identified from fungi. The isolation of the xanthone ravenelin 28 from *Helminthosporium ravenelii*^6^ and the grisandienes geodin 19 and erdin 20 from *Aspergillus terreus*,^7^ for example, were both reported by Raistrick and colleagues in 1936, although the structures of the grisandienes were not fully solved until 1958.^8^ The well-known dimeric ergochromes were isolated and studied by many groups throughout the 20^th^ century, and by 1969 the structures of secalonic acids A 39, B 40 and C, and the closely related dimeric ergochrysins A and B and ergoflavin had been determined, along with key aspects of their biosynthesis.^9^

The isolation and structural elucidation of various related compounds and intermediates, coupled with feeding studies using isotopically labelled compounds, gradually allowed potential biosynthetic routes to emerge. In plants anthraquinones are biosynthesised either *via* the acetate-malonate (*i.e.* polyketide) pathway or *via* the chorismate/*O*-succinylbenzoic acid pathway, but filamentous fungi only use the former.^10^ In 1958 Gatenbeck demonstrated that in *Talaromyces islandicus* (previously *Penicillium islandicum*), emodin 1 is formed from condensation of 8 molecules of acetate,^11^ with similar feeding studies demonstrating acetate incorporation into various related compounds.

As biosynthetic routes were being proposed, biosynthetic relationships also become apparent. A shared biosynthetic pathway for the xanthone ergochromes (*e.g.*39) and sulochrin 15*via* anthraquinone intermediates was proposed as early as 1966.^12^ Proof of emodin 1 as an intermediate in the biosynthesis of secalonic acids was then provided *via* feeding studies using either ^14^C or tritium labelled emodin 1.^9^ In the same year, it was shown that ^14^C labelled questin 9, prepared by enzymatic methylation of emodin 1, was converted into sulochrin 15.^13^ The link between grisandienes such as geodin 19 and the anthraquinones was proven by Fujimoto *et al.*,^14^ who fed [UL-^14^C] emodin 1 to *A. terreus* and demonstrated that it was incorporated into geodin 19. In significant work of the 1980s and 90s, Fujii and colleagues identified various key enzymatic activities in the pathway to geodin 19, largely through the use of cell-free assays.^15–19^

More recently, the advent of the genomic era – allowing the identification of gene clusters and subsequent gene function investigations – has generated significant insights into the biosynthesis of the emodin family of compounds. Bioinformatic analyses, the generation and analysis of mutant strains, heterologous expression and *in vitro* assays have combined to confirm certain proposals whilst amending others. Although various biosynthetic pathways have been proposed, the premise of this review is that, as a biosynthetic family of natural compounds arising from largely homologous biosynthetic gene clusters (BGCs), clear experimental evidence from one pathway can be extrapolated to others – therefore providing a universal overview of their biosynthesis. The pathways proposed in this review are therefore the authors' conclusions based on all available current evidence.

There have been excellent reviews on many of the compound classes discussed here, including anthraquinones^4,20–22^ and xanthones,^23–25^ but relatively few of these focus specifically on fungal compounds, and none – to our knowledge – bring together our current understanding of the entire emodin-related biosynthetic family. Additionally, there have been major advances in recent years, for example in understanding key aspects of xanthone biosynthesis, such as formation of the xanthone core, and stereochemical control.

**Fig. 2:**
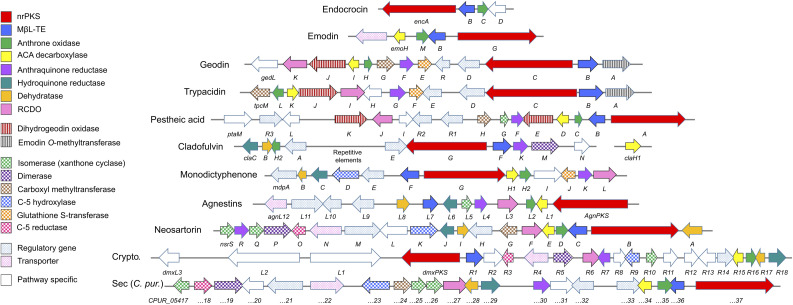
Characterised biosynthetic gene clusters (BGCs) encoding compounds belonging to the ‘emodin family’ of fungal natural products. See Table 1 for predicted functions of pathway specific genes. Crypto. = Cryptosporioptides. ACA = atrochrysone carboxylic acid. RCDO = ring-cleaving dioxygenase.

This review will be divided into sections discussing what can be considered the core biosynthetic pathway to key intermediates such as emodin 1 and chrysophanol 2, with specific branches from that pathway being discussed separately. A bioinformatic analysis, identifying homologues within the characterised pathways will be referred back to throughout the review, and is summarised in Table 1 and Fig. 2.

**Table 1:** Genes located within the identified gene clusters of endocrocin 5 (*Enc*), emodin 1 (*Emo*), geodin 19 (*Ged*), trypacidin 21 (*Tpc*), pestheic acid 22 (*Pta*), cladofulvin 11 (*Cla*), monodictyphenone 12 (*Mdp*), the agnestins: *e.g.*30, 31 (*Agn*), the cryptosporioptides: *e.g.*34–36 (*Dmx*), the secalonic acids from *Claviceps purpurea*39, 40 (*Cpur*) and neosartorin 38 (*Nsr*). Genes on each row are considered likely to encode functionally homologous proteins due to high% protein identity. SDR = short-chain dehydrogenase/reductase

	PREDICTED FUNCTION	*Enc* ^26^	*Emo* ^55^	*Ged* ^27^	*Tpc* ^28,29^	*Pta* ^30^	*Cla* ^31,32^	*Mdp* ^33^	*Agn* ^34^	*Dmx* ^32^	*Cpur* ^32,35^	*Nsr* ^36^
Steps to emodin	Group V nrPKS	*encA*	*emoG*	*gedC*	*tpcC*	*ptaA*	*claG*	*mdpG*	*agnPKS1*	*dmxPKS*	*05437*	*nsrB*
	MβL-TE	*encB*	*emoB* a	*gedB*	*tpcB*	*ptaB*	*claF*	*mdpF*	*agnL7*	*dmxR1*	*05436*	*nsrC*
	Decarboxylase	*encD*	*emoH*	*gedI*	*tpcK*	*ptaD*	*claH1*	*mdpH1*	*agnL1*	*dmxR15*	*05434*	*nsrE*
	Anthrone oxidase	–	*emoM*	*gedH*	*tpcL*	*ptaC*	*claH*	*mdpH2*	*agnL2*	*dmxR16*	*05435*	*nsrD*
Anth. reductase	Oxidoreductase	–	–	*gedF*	*tpcG*	*ptaF*	*claK*	*mdpK*	*agnL4*	*dmxR7*	*05430*	*nsrR*
Steps to chrysophanol	Reductase	–	–	–	–	–	*claC*	*mdpC*	*agnL6*	*dmxR18*	*05429*	*nsrJ*
	Dehydratase	–	–	–	–	–	*claB*	*mdpB*	*agnL8*	*dmxR17*	*05428*	*nsrI*
RCDO	Ring cleaving dioxygenase	–	–	*gedK*	*tpcI*	*ptaJ*	–	*mdpL*	*agnL3*	*dmxR6*	*05427*	*nsrF*
Carboxyl methyltransferase	*O*-Methyltransferase	–	–	*gedG*	*tpcM*	*ptaH*	–	–	–	–	*05424*	*nsrG*
Grisandiene only	Glutathione S-transferase^b^	–	–	*gedE*	*tpcF*		–	–	–	–	–	–
	(Emodin) *O*-methyltransferase	–	–	*gedA*	*tpcA*	–	–	–	–	–	–	–
Gris. And pes. only	Dihydrogeodin oxidase	–	–	*gedJ*	*tpcJ*	*ptaE ptaK*	–	–	–	–	–	–
Dimerase	P450	–	–	–	–	–	*claM*	–	–	*dmxR5*	*05419*	*nsrP*
C-5 hydroxylase	Monooxygenase	–	–	–	–	–	–	*mdpD*	–	*dmxR9*	*05423*	*nsrK*
C-5*S* reductase	SDR	–	–	–	–	–	–	–	–	*dmxR3*	*05418*	*nsrO*
Xanthone cyclase	SnoAL domain protein	–	–	–	–	*ptaG*	–	–	*agnL5*	*dmxR10*	*05417*	*nsrS*
											*05425*	*nsrQ*
											*05426*	
Enc only	2-Oxoglutarate oxidoreductase	*encD*	–	–	–	–	–	–	–	–	–	–
Geo only	Sulochrin halogenase	–	–	*gedL*	–	–	–	–	–	–	–	–
Tpc only	*O*-methyltransferase	–	–	–	*tpcH*	–	–	–	–	–	–	–
Pes only	*O*-methyltransferase	–	–	–	–	*ptaI*	–	–	–	–	–	–
	Oxidoreductase	–	–	–	–	*ptaL*	–	–	–	–	–	–
	Isosulochrin halogenase	–	–	–	–	*ptaM*	–	–	–	–	–	–
Cla only	Oxidoreductase	–	–	–	–	–	*claN*	–	–	–	–	–
Mdp only	Acyl-CoA synthase	–	–	–	–	–	–	*mdpI*	–	–	–	–
	Glutathione S-transferase^b^	–	–	–	–	–	–	*mdpJ*	–	–	–	–
Cry only	Hemerythrin-like domain protein	–	–	–	–	–	–	–	–	*dmxR11*	–	–
	SDR	–	–	–	–	–	–	–	–	*dmxR12*	–	–
	*O*-Acetyltransferase	–	–	–	–	–	–	–	–	*dmxR13*	–	–
	Cytochrome P450	–	–	–	–	–	–	–	–	*dmxL3*	–	–
	hrPKS	–	–	–	–	–	–	–	–	*dmxL2*	–	–
	Acyl-CoA carboxylase	–	–	–	–	–	–	–	–	*dmxL1*	–	–
	SDR	–	–	–	–	–	–	–	–	*dmxR8*	–	–
	Putative transferase	–	–	–	–	–	–	–	–	*dmxR2*	–	–
Sa specific	Hypothetical	–	–	–	–	–	–	–	–	–	*05420*	–
	Monooxygenase	–	–	–	–	–	–	–	–	–	*05431*	–
	SDR (C-5*R* reductase)	–	–	–	–	–	–	–	–	–	–	–
Neo only	*O*-Acetlytransferase	–	–	–	–	–	–	–	–	–	–	*nsrL*
Non catalytic	AflR-like transcription factor	–	–	*gedR*	*tpcE*	*ptaR2*	*claE*	*mdpE*	*agnL10*	*dmxR14*	*05433*	*nsrA*
	AflS-like coactivator	–	–	*gedD*	*tpcD*	*ptaR1*	*claA*	*mdpA*	*agnL9*	–	*05432*	*nsrH*
	Transcription factor	–	–	–	–	*ptaR3*	–	–	*agnL11*	–	*05421*	*nsrM*
	MFS transporter	–	41 650.1	–	–	–	–	–	*agnL12*	*dmxR4*	*05422*	*nsrN*

a EmoB was not identified in the original analysis and is missing from the MiBIG database. It was identified during the writing of this review, through softberry analysis of the genomic sequence.

b Putative glutathione S-transferases are listed in two places (Grisandiene only, and mdp only) due to demonstrating protein identity below 25%, suggesting a lack of functional homology.

## 2 The core anthraquinone pathway – generating emodin and chrysophanol

### 2.1 Emodin biosynthesis

**Scheme 1:**
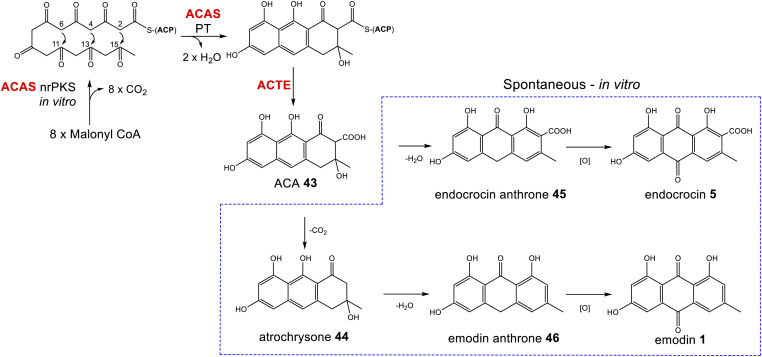
ACA 43 biosynthesis *in vitro*. Spontaneous decarboxylations, dehydrations and oxidations can generate a range of ACA-derived compounds.^40^

Phylogenetic analyses have classified fungal non-reducing polyketide synthases (nrPKSs) as belonging to eight distinct and evolutionarily related groups (I–VIII).^37^ Group V nrPKSs, which are responsible for the biosynthesis of various aromatic fungal secondary metabolites, including aflatoxin, asperthecin and emodin 1, are rather unusual as they all lack a thioesterase (TE) or Claisen-like cyclase (CLC) domain for release, and require the action of a discrete hydrolase, specifically a metallo-β-lactamase-type thioesterase (MβL-TE).^38^ The evolution and diversity within group V was recently explored by Keller and colleagues, who further categorized group V nrPKS enzymes into three evolutionarily and biochemically linked subsets that produce polyketides with differing chain lengths and cyclization patterns.^39^ The emodin family of natural products are all produced by synthases from subset V1; octaketide synthases that catalyse an initial “C6–C11 cyclisation”. This produces the tricyclic core of atrochrysone carboxylic acid 43 (ACA – Scheme 1); the first enzyme-free intermediate in the biosynthetic pathway to emodin 1. The first ACA synthase (ACAS) to be identified was from *Aspergillus terreus*,^40^ later shown to belong to the geodin 19 gene cluster (encoded by *gedC*).^27^

As mentioned, ACAS lacks a release domain, but otherwise consists of typical nrPKS domains; namely SAT (starter unit:ACP transacylase), KS (β-ketosynthase), AT (acyltransferase), PT (product template) and ACP (acyl carrier protein). A fragment of ACAS was first identified *via* degenerate PCR in a study designed to disrupt sulochrin 15 biosynthesis in *A. terreus* as an undesirable co-metabolite of lovastatin.^41^ Subsequent identification of the full gene, followed by *in vitro* assays and heterologous expression in *A. oryzae* characterised the products of the PKS and identified the role of the MβL-TE thioesterase – called atrochrysone carboxyl ACP thioesterase (ACTE) – in product release (Box 1).^40^

It has been proposed that malonyl-CoA, rather than acetyl-CoA, is the starter unit as well as the extender unit for ACAS enzymes. This is unusual in fungal polyketides, but has some precedent, such as PKS4 from *Fusarium fujikuroi*^42^ (previously *Gibberella fujikuroi*) and potentially the T4HN PKS from *Colletotrichum lagenariumi*,^43^ although there is ongoing debate regarding the details of T4HN biosynthesis. *In vitro* assays for ACAS, combining [1-^14^C]acetyl-CoA as the starter substrate, with unlabelled malonyl-CoA failed to produce any radio-labelled products, implying that acetyl-CoA is not directly involved in ACA biosynthesis. Additionally, a bioinformatic analysis of the SAT domain of ACAS found that it lacks the active-site cysteine of the GXCXG motif identified by Townsend and coworkers.^44^ It was concluded from this that the SAT domain in these enzymes are likely to lack any acyl transferase activity and instead the acyl transferase (AT) domain of the nrPKS may load all malonate building blocks, including the starter unit. Interestingly, in the bacterial type II iterative actinorhodin PKS, it has been shown that the holo-ACP is capable of catalytic self-malonylation in the presence of malonyl-CoA, with subsequent decarboxylation generating acetyl ACP as the “starter unit”.^45^ In this system, the MCAT (malonyl-CoA:*holo*-ACP transacylase) has been reported as showing no measurable acetyl–transferase activity, and is not an absolute requirement for polyketide biosynthesis.^46^

**Fig. 3:**
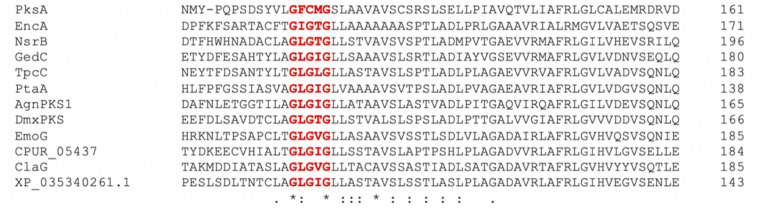
An alignment of pksA; the aflatoxin PKS from *A. flavus* with all known fungal ACAS enzymes shows that the loss of the GXCXG SAT domain motif appears to be a universal feature of ACAS enzymes. The putative rugulosin PKS (XP_035340261.1) also contains the C to G mutation.

**Fig. 4:**
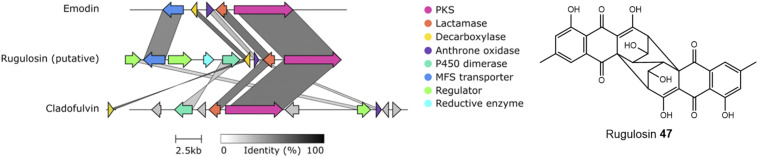
A putative rugulosin 47 BGC can be identified within the publicly available genome of *T. rugulosus* (GeneIDs: 55988671–55988678). This cluster contains all of the genes required for emodin 1 biosynthesis, and also other key genes, such as a homologue to the confirmed anthraquinone dimerase from the cladofulvin 11 BGC. Comparison conducted using Clinker.^48^

An alignment of the PKSs discussed in this review and shown in Table 1 shows that the cysteine to glycine mutation observed for the *A. terreus* ACAS appears to be universal in this family of nrPKSs (Fig. 3), but whether this mutation truly leads to a loss of function is yet to be experimentally explored. Previous studies identified acetyl-CoA as the starter unit for the dimeric anthraquinone rugulosin 47 (Fig. 4), due to the retention of three deuterium atoms at the C-6 methyl group when fed with [2-^2^H_3_,2-^13^C]acetate.^49^ Rugulosin 47 is thought to be derived from emodin 1, and indeed, interrogation of the publicly available genome of *Talaromyces rugulosus* identified a putative rugulosin gene cluster (GeneIDs: 55988671–55988678; Fig. 4). This locus encodes all of the expected enzymes for the biosynthesis of 47*via*1, including the ACAS/MβL-TE, a decarboxylase and anthrone oxidase, reductive enzymes and a P450, which could catalyse the required 5,5-oxidative dimerisation. The ACAS at this locus (accession number: XP_035340261.1) aligns well with all other nrPKSs from emodin clusters and also contains the cysteine to glycine mutation (Fig. 3), raising the possibility that this mutation does not lead to a loss of function. Feeding [2-^2^H_3_,1-^13^C]acetate to confirmed producers of emodin 1 and related compounds, where the identity of the PKS is confirmed, could serve to conclusively answer this key question. This is another clear example of where simple labelling studies can serve to challenge assumptions based on genetic analyses alone, sometimes made with insufficient regard to chemical and biochemical requirements.

**Box 1**

The atrochrysone carboxylic acid synthase (ACAS) and atrochrysone carboxyl ACP thioesterase (ACTE) from *Aspergillus terreus* were first characterised by Awakawa and colleagues *via in vitro* assays.^40^ This work highlighted the inherent instability of atrochrysone carboxylic acid 43 (ACA). *In vitro* assays with only the nrPKS(ACAS)/MβL-TE(ACTE) pairing produced a range of compounds including atrochrysone 44, endocrocin anthrone 45, endocrocin 5, emodin anthrone 46 and emodin 1 (Scheme 1). β-Keto acids are generally vulnerable to spontaneous decarboxylation due to a low energy path for decarboxylation *via* the enol of the product ketone.^47^ Such a decarboxylation leads to the conversion of ACA 43 to atrochrysone 44. Spontaneous dehydration of atrochrysone 44 can then produce emodin anthrone 46. Conversely, when aromatisation by dehydration of ACA 43 occurs initially, producing endocrocin anthrone 45, the lack of a ketone at the β position then makes decarboxylation thermodynamically unfavourable. Finally, the presence of endocrocin 5 and emodin 1 in the *in vitro* assays demonstrates that oxidation of anthrones to produce anthraquinones can also happen spontaneously in *in vitro* systems. A similar range of compounds was produced when the nrPKS/MβL-TE combination from the cladofulvin 11 gene cluster (*claG*/*claF*) were heterologously expressed in *A. oryzae*, although it is always possible that in such *in vivo* systems native enzymes act on heterologous compounds.^31^

A series of steps including a decarboxylation, dehydration and oxidation are required to convert ACA 43 – the product of the nrPKS–to the key anthraquinone intermediate, emodin 1. A range of biosynthetic routes can be found in the literature, with variations in the order of events, the identity of the intermediates and whether specific steps are simply spontaneous or catalysed by dedicated pathway enzymes. Multiple factors have made the details of emodin 1 biosynthesis in fungi relatively difficult to elucidate; in particular: (i) mis-annotations in the early bioinformatic analyses of gene clusters meant that genes encoding a decarboxylase and anthrone oxidase were not initially identified,^33,39^ and (ii) ACA 43 and related compounds are relatively unstable (Box 1), meaning that various compounds – including emodin itself – can come about spontaneously. Such instability also leads to the frequent accumulation of shunt products rather than genuine intermediates, making pathway elucidation more difficult.

To resolve the historical inconsistencies in the literature, firstly we will review the identification and characterisation of specific enzyme activities involved in the biosynthesis of emodin 1. Secondly, we will summarise arguments for the order of events being decarboxylation and dehydration to emodin anthrone 46 followed by oxidation to the anthraquinone.

The first clear evidence of a catalytic decarboxylase enzyme came from investigations into the monodictyphenone 12 pathway.^50^ The deletion of a fragment of the monodictyphenone (*mdp*) gene cluster, then annotated as *mdpH*, led to a significant reduction in monodictyphenone 12 titres and the accumulation of endocrocin 5, suggesting a putative role for this genomic region in decarboxylation. Later reannotation of *mdpH* determined that this region in fact contains two genes, now renamed *mdpH1* and *mdpH2*, and it is *mdpH1* that encodes the decarboxylase.^39^ Investigations into the trypacidin 21 pathway identified and deleted the decarboxylase-encoding *tpcK* and similarly observed an accumulation of endocrocin 5.^29^ In the absence of the decarboxylase, ACA 43 is thought to undergo a spontaneous dehydration to give endocrocin anthrone 45, which is then oxidised, likely by the action of an anthrone oxidase (see later discussion), to the anthraquinone endocrocin 5. This supposition is supported by the identification of a dedicated endocrocin gene cluster in *Aspergillus fumigatus*, which contains the nrPKS (*encA*)/MβL-TE (*encB*) pairing as well as an anthrone oxidase (*encC*) but no decarboxylase.^26^

The first heterologous expression experiments to examine the role of the decarboxylase came from work on the cladofulvin 11 biosynthetic pathway of *Cladosporium fulvum*. Heterologous expression of various genes from the cladofulvin gene cluster in *Aspergillus oryzae*, combined with time course data, suggested that spontaneous dehydration of ACA 43 occurs faster than decarboxylation, directing the pathway towards endocrocin 5 rather than emodin 1.^31^ Thus, the action of a decarboxylase is needed to efficiently produce 1. The expression of only the nrPKS (ClaG) and MβL-TE (ClaF) produced a range of compounds similar to those seen previously in *in vitro* assays;^40^*i.e.* atrochrysone 44 and endocrocin 5 as well as small amounts of emodin anthrone 46 and emodin 1. When the decarboxylase ClaH was also expressed, 1 was produced as the single major compound, which is perfectly consistent with a catalytic decarboxylation being responsible for directing biosynthesis towards emodin 1 production. Homologues to the above decarboxylase enzymes are encoded by all of the identified emodin-producing gene clusters, including the cluster for pestheic acid 22, where the pathway proposed in the literature included spontaneous decarboxylation.^30^

Enzymes catalysing the oxidation of anthrones to produce anthraquinones such as emodin 1 have been known for some time. The identification of an enzyme named emodinanthrone oxygenase from *Aspergillus terreus* was reported as early as 1991 by Fujii and colleagues, and shown through incubation with ^18^O_2_ to fix an atom of molecular oxygen at the C-10 position of emodin anthrone 46.^18^ The purification and further characterisation of emodinanthrone oxygenase was reported in 1995,^51^ but the gene encoding this enzyme was not identified until 2013.^52^ A gene encoding a bacterial anthrone oxidase, AknX, was reported in 2002 and proposed to catalyse the production of aklanonic acid from its anthrone precursor,^53^ and this was followed by the identification of the first fungal gene encoding an anthrone oxidase, called HypC, in 2010. HypC is involved in aflatoxin biosynthesis in *Aspergillus parasiticus*, and was shown, through both gene deletions and *in vitro* assays, to catalyse the conversion of norsolorinic acid anthrone (NAA) to norsolorinic acid (NA).^54^ Interestingly, in addition to catalysing the oxidation of their pathway specific anthrones, both AknX and HypC can catalyse the conversion of emodin anthrone 46 to emodin 1, showing their relatively relaxed specificity, a feature that may aid the evolution of novel anthraquinones. The gene encoding the true emodinanthrone oxygenase, GedH, from the geodin pathway, was then identified based on homology to *hypC*,^52^ having previously been missed due to the mis-annotation of a neighbouring gene.

The original mis-annotation of *mdpH* discussed above meant that the anthrone oxidase in the monodictyphenone 12 pathway was also originally missed, leading to the suggestion that such an oxidation occurs spontaneously. As shown by the *in vitro* work of Awakawa *et al.*, (Box 1) anthrone oxidation can indeed occur spontaneously, and this is further demonstrated by the fact that deletion of the anthrone oxidase *hypC* still allowed the accumulation of anthraquinones and later pathway products in *Aspergillus parasiticus*, albeit at reduced titres.^54^*In vitro* assays have also shown that norsolorinic acid anthrone will spontaneously convert to norsolorinic acid in 24 hours in the absence of any catalytic enzyme, but this conversion is almost complete in only 1 hour in the presence of HypC.^54^ The annotation of the monodictyphenone 12 gene cluster was later corrected and the monodictyphenone anthrone oxidase is now known as MdpH2. MdpH2 homologues can be found in all gene clusters known to produce emodin 1 and related compounds (Table 1), including the cladofulvin 11 cluster, where it was missed in the initial publication,^30^ but later identified through comparative bioinformatics.^31,32^ Hence, while it is reasonable to posit that anthrone oxidations can occur spontaneously, the efficient production of emodin 1 and related compounds is universally supported by the action of a dedicated anthrone oxidase.

Thus, the fact that emodin 1 can be produced from ACA 43*via* spontaneous dehydrations, decarboxylations and autoxidations (Box 1), does not mean that these steps simply occur spontaneously in the biosynthetic pathways. The two key conversions required to direct ACA 43 towards emodin 1 are a decarboxylation and oxidation, and enzymes catalysing both steps are now known and can be found in all gene clusters belonging to the emodin family of fungal natural products (Table 1). In keeping with the minimal emodin gene set being an nrPKS, MβL-TE, decarboxylase and anthrone oxidase, a dedicated emodin gene cluster was recently identified from *Escovopsis weberi* consisting of exactly those genes.^55^*Escovopsis weberi* is a specialised pathogen that infects the fungal crops farmed by leaf-cutter ants. This species is thought to use emodin 1 itself, rather than any derivatives, as an antibacterial and antifungal agent in chemical warfare with both the bacterial (*Streptomyces* and *Pseudonocardia*) and fungal (*Leucoagaricus gongylophorus*) symbionts of leaf-cutter ants.^55,56^ The published gene cluster (MiBIG: BGC0001583) missed the discrete MβL-TE, but reanalysis of the genomic sequence during the writing of this review, identified a predicted protein with 63.3% homology with ACTE, the characterised MβL-TE from *Aspergillus terreus*.

The identification of anthrone oxidase and decarboxylase enzymes does not necessarily pin down the order of events in emodin 1 biosynthesis. The original proposal for the biosynthesis of monodictyphenone 12 in 2009,^50^ and the later proposal for the pestheic acid 22 pathway^30^ both show dehydration of ACA 43 leading to endocrocin anthrone 45, which is first oxidised to endocrocin 5 then finally decarboxylated to give emodin 1. The putative identification of endocrocin 5 as an intermediate in the pathways may well have been, in part, due to the isolation of endocrocin 5 from producing strains. However, as previously discussed, 5 is likely to be a shunt product in these pathways, and this order of events is highly unlikely for various reasons. Firstly, decarboxylation of 5 to 1 is considered to be thermodynamically unfavourable (see Box 1).^33,40^ Secondly, early feeding studies conducted by Steglich and colleagues found that in *Dermocybe* fungi, radiolabelled endocrocin 5 was not incorporated into emodin 1, even though it was successfully incorporated into endocrocin derivatives retaining the carboxyl group such as dermolutein 7 and dermorubin 8.^57^ More recently, gene deletions from the trypacidin 21 pathway have suggested that decarboxylation is likely to occur prior to anthrone oxidation based on the fact that the double decarboxylase/oxidase mutant (Δ*tpcK*,Δ*tpcL*) has the exact same metabolic profile as the single decarboxylase mutant (Δ*tpcK*). In this latter study,^29^ Throckmorton and colleagues proposed that endocrocin 5 is detected as a shunt product in all gene clusters that produce emodin *via* an nrPKS, MβL-TE, anthrone oxidase and decarboxylase due to incomplete activity of the decarboxylase.

**Scheme 2:**
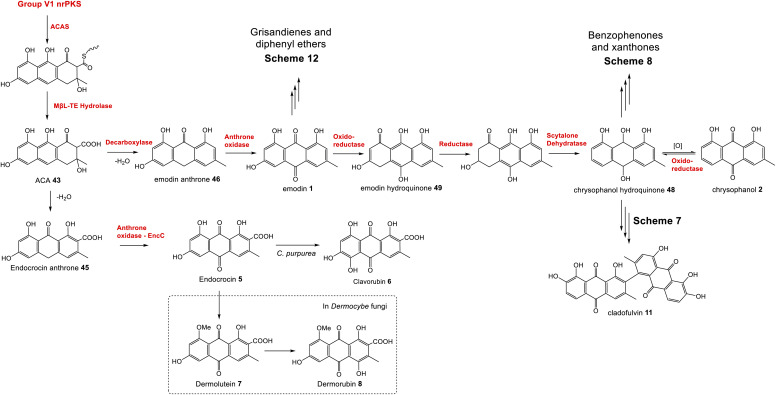
The core biosynthetic pathway of the emodin family of natural products proceeds *via* atrochrysone carboxylic acid (ACA) 43, the anthraquinones emodin 1 and chrysophanol 2, and their corresponding hydroquinones.

To summarise, although both emodin 1 and endocrocin 5 can be produced from ACA 43*via* spontaneous dehydrations, decarboxylations and autoxidations, the enzymes in the emodin 1 pathway of filamentous fungi direct the biosynthetic pathway towards 1 through the action of pathway specific enzymes. A concerted decarboxylation-elimination of ACA 43 – catalysed by an MdpH1-like decarboxylase – gives emodin anthrone 46, which then undergoes oxidation by an MdpH2-like anthrone oxidase to produce emodin 1 (Scheme 2).

### 2.2 Emodin to chyrosophanol

A key step in the biosynthetic pathway of many emodin related compounds is the deoxygenation of emodin 1 to produce the anthraquinone chrysophanol 2 along with its reduced form, chrysophanol hydroquinone 48. Many natural products that are emodin-derived lack the 6-hydroxy group, and there is now a general consensus that reductive deoxygenation of 1 is a shared early step in their biosynthesis, and that this occurs prior to the key ring cleavage step required for the biosynthesis of core structures such as benzophenones and xanthones.

The first key evidence for chrysophanol 2 (or its reduced form, chrysophanol hydroquinone 48) as a biosynthetic intermediate came in 1980, when Franck *et al.* investigated potential intermediates in the biosynthesis of secalonic acid D 41 (ergochrome EE) by using competitive incorporation.^58^ [U-^3^H]emodin was fed to cultures of *Penicillium oxalicum* along with a ^14^C-labelled anthraquinone; either emodin 1, chrysophanol 2, islandicin 3 or catenarin 4. Chrysophanol 2 was shown to be incorporated 3.6 times more readily than 1, leading to the conclusion that biosynthesis proceeds from emodin 1*via* chrysophanol 2. No incorporation for catenarin 4 was observed, and incorporation of islandicin 3 was 12 times lower than 2, leading to the conclusion that 3 is principally a shunt product. Further evidence then came from a cell-free system of the secalonic acid producer *Phoma terrestris*, which was used to enzymatically convert labelled emodin 1 to chrysophanol 2 in the presence of NADPH.^59,60^ Isotopic feeding studies have also demonstrated that 2 labelled with deuterium in the methyl group is specifically incorporated into the prenylated xanthones shamixanthone 32 and tajixanthone 33, produced by *Aspergillus variecolor*.^61,62^

The more recent analysis of related biosynthetic pathways through gene cluster analysis and gene deletions has now confirmed that deoxygenation of emodin occurs at an early stage in many pathways. Deletion of genes encoding the ring cleaving oxygenase (originally assigned as putative Baeyer–Villiger monooxygenases, but see Section 3.1 below) from the agnestin BGC of *Paecilomyces variotii* (*agnL3*),^34^ the cryptosporioptide BGC of *Cryptosporiopsis* sp. (*dmxR6*),^32^ the neosartorin BGC of *Aspergillus novofumigatus* (*nsrF*)^36^ and indeed the monodictyphenone BGC (*mdpL*),^63^ all led to an accumulation of chrysophanol 2. In the case of the *nsrF* mutant, some neosartorin 38 was still produced but this may be explained by the presence of a gene encoding a homologue to NsrF (approximately 40% protein sequence identity) elsewhere in the genome.

**Scheme 3:**
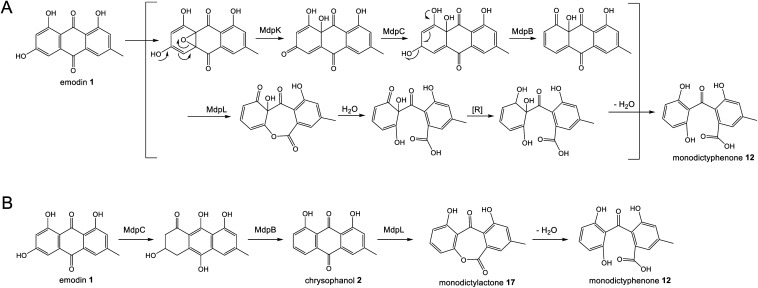
Previous proposals for the pathway from emodin 1 to monodictyphenone 12. Please see Section 3.1 and Scheme 5B for the most likely pathway based on recent work by Lu and colleagues.^75^ (A) Original monodictyphenone 12 pathway proposed by Chiang *et al.*^33^ (B) Proposal by Simpson in 2012,^65^ which reconciled all available evidence at the time, including the identification of chrysophanol 2 as a likely intermediate.

Original proposals for the biosynthesis of monodictyphenone 12 – and by extension the related prenylated xanthones – excluded chrysophanol 2 as an intermediate. The *mdp* gene cluster was first published in 2009 after the deletion of *cclA* – a gene involved in epigenetic regulation in *A. nidulans* – upregulated the cluster and led to the production of emodin 1, emodin derivatives and monodictyphenone 12.^50^ In 2010 Chiang *et al.*^33^ reported the generation and analysis of various mutant strains and proposed a biosynthetic pathway, based largely on the aflatoxin pathway of the time,^64^ which involved the epoxidation of emodin 1 followed by an isomerisation, ketoreduction and dehydration, a Baeyer–Villiger oxidation to give a lactone and finally hydrolysis, reduction and spontaneous aromatisation to produce monodictyphenone 12 (Scheme 3A). However, there were various inconsistencies in this proposal, not least the accumulation of chrysophanol 2 upon deletion of the oxygenase *mdpL*^63^ and the identification of 2 as an intermediate in the prenylated xanthones,^61,62^ which are produced by the same biosynthetic pathway as monodictyphenone 12.

A more recent publication on the biosynthesis of secalonic acids by *C. purpurea*,^35^ shows a pathway that is homologous to the original monodictyphenone 12 pathway – *i.e. via* the epoxidation of emodin 1 – but again this is inconsistent with the identification of chrysophanol 2 as an intermediate in secalonic acid biosynthesis in multiple species.^58,60^

In 2012 an argument for an alternative route, where monodictyphenone 12 biosynthesis proceeds *via* the deoxygenation of emodin 1 to chrysophanol 2 prior to oxidative ring cleavage (Scheme 3B) was presented.^65^ Although this proposal succeeded in reconciling the isotopic labelling studies, likely intermediates, and genetic understanding of the time, it now appears that certain details were incorrect and a recent study may have finally revealed the true pathway. As discussed in detail in Section 3.1 (also, see Scheme 5A and B), although key aspects of this newly proposed pathway differ from the previous proposal,^65^ it also supports the deoxygenation of emodin as an early step, occurring prior to ring cleavage and diversification of the core structure.

Although the pathway to monodictyphenone 12 proposed by Chiang *et al.*^33^ (Scheme 3A) is now know to be incorrect, the reductase and dehydratase enzymes involved in the removal of the C6-hydroxy group were correctly identified. MpdC was identified as a likely reductase due to strong amino acid identity with various known enzymes including tetrahydroxy-naphthalene reductase (T_4_HNR), which is involved in the key deoxygenation steps in melanin biosynthesis,^66^ and the versicolorin reductase AflM from the aflatoxin biosynthetic pathway.^67^ MdpB was identified as a putative dehydratase due to high sequence homology with the well characterised scytalone dehydratase,^68^ also involved in melanin biosynthesis. The deletion of either *mdpC* or *mdpB* from *A. nidulans* led to the accumulation of emodin 1 and various oxidised emodin analogues, which is compatible with the role of these genes in the deoxygenation of emodin.^33^

**Scheme 4:**
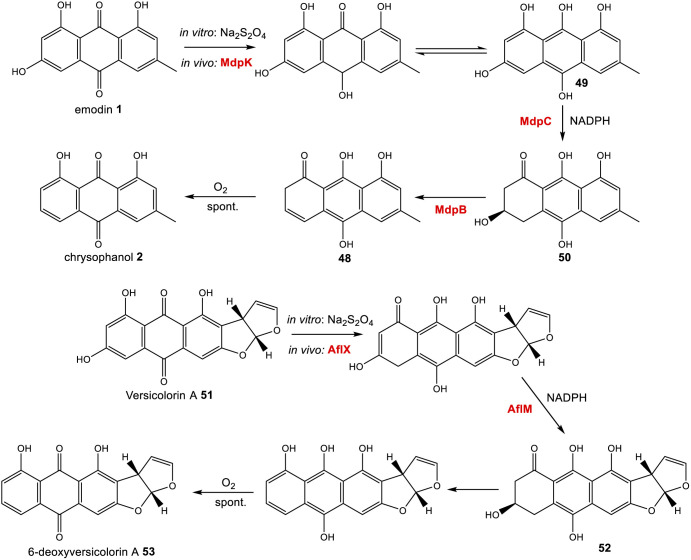
Prior reduction of anthraquinones 1 and 51 is required before phenol reduction. *In vitro* this has been achieved using Na_2_S_2_O_4_. *In vivo*, this is thought to be catalysed by the homologous oxidoreductases MdpC and AflX.^34^

Further evidence for the roles of MdpB and MdpC comes from heterologous expression experiments as well as extensive *in vitro* assays. The expression of the cladofulvin 11 homologues, ClaB and claC, in *A. oryzae* created a strain capable of producing chrysophanol 2 when fed with emodin 1, although the turnover was minimal, with only trace amounts of 2 being produced.^31^ Elegant chemoenzymatic work by Müller and colleagues involving *in vitro* assays confirmed that the exact function of MdpC is as an anthrol reductase, acting on the corresponding anthrol – produced *in vitro* by prior dithionite reduction – rather than the anthraquinone. This led to a newly proposed pathway from emodin 1 to chrysophanol 2, where 1 must first be reduced to the tautomeric forms of emodin hydroquinone 49, which can then undergo a phenol reduction catalysed by MdpC, followed by a dehydration catalysed by MdpB and finally a spontaneous autoxidation to give 2 (Scheme 4).^69^ The requirement for initial reduction to a hydroquinone prior to the action of an anthrol reductase is consistent with studies of melanin biosynthesis. Tetrahydroxy- and trihydroxy-naphthalenes exist mainly as keto–enol tautomers in aqueous buffer (*e.g.* 1,3,8-trihydoxynapthalene^70^) and are readily reduced both chemically and enzymatically, suggesting that keto–enol tautomerism – as is pronounced in anthrahydroquinones – may be a requirement for enzymatic reduction. This led Müller and co-workers to suggest that the emodin hydroquinone tautomers may actually be the dominant forms of emodin 1*in vivo* due to the cell being in a highly reduced state. The most intriguing outcome of this study was that MdpC does not accept emodin 1, as was previously thought. Rather, *in situ* reduction of 1 by sodium dithionite was required before MdpC could generate the hydroxyketone 50.^69^ A similar chemical reduction of versicolorin A 51 was later shown to be necessary before the action of the anthraquinone reductase AflM in the aflatoxin pathway of *Aspergillus parasiticus*.^71^

More recently characterised reductases with homology to MdpC are 17β-HSDcl from *Curvularia lunata*^72^ and ARti and ARti-2 from *Talaromyces islandicus.*^73,74^ These anthrol reductases were all found to have a versatile substrate range *in vitro*, including steroids such as estrone and polyhydroxynaphthalenes such as T_4_HN, as well as anthrols. The location of the genes encoding these enzymes within Group V1 nrPKS gene clusters suggests that their physiological functions *in vivo* are likely to be homologous to MdpC, and as with MdpC both have been shown to require an initial reduction of emodin 1 to emodin hydroquinone 49 prior to catalysing the phenol reduction.

There is now evidence that reduction from anthraquinone to hydroquinone is likely to be enzymatically controlled. In the neosartorin 38 gene cluster, an SDR encoded by *nsrR* was proposed as catalysing the required reduction of emodin 1, based on the fact that this was the only reductive enzyme of unknown function encoded by the BGC that also had homologues in the cladofulvin 11 and shamixanthone 32 clusters.^36^ Published in the same month as the neosartorin paper, the enzymatic basis for this reduction was probed by Simpson and colleagues during investigations into the biosynthesis of the agnestins (*e.g.*30 and 31).^34^ AgnL4 from the agnestin pathway, and indeed NsrR, are homologues to MdpK from the monodictyphenone pathway, for which multiple potential roles have been proposed. It was initially suggested that MdpK played a role in rearrangement of the epoxide intermediate in the conversion of emodin 1 to monodictyphenone 12 (Scheme 3A above). This was based on homology to AflX and its proposed role in the anthraquinone (VA) to xanthone (DMST) conversion in the aflatoxin pathway,^64^ but as argued by Simpson, it is unlikely that rearrangement of such an epoxide intermediate is necessary in monodictyphenone 12 biosynthesis.^34^ MdpK was later suggested as a thiolester reductase, producing the aldehyde of monodictyphenone 12 in the pathway to the prenylated xanthones.^65^ However, deletion of *agnL4* and the resulting substantial accumulation of emodin 1 in the mutant strain has now identified AgnL4 as the likely missing link in chrysophanol 2 biosynthesis, by catalysing the required reductive reaction prior to phenol reduction.^34^

Reviewing the gene deletions conducted for the *mdp* BGC,^33,63^ disruption of MdpK does lead to an accumulation of emodin 1 and emodin analogues, which is consistent with emodin being the substrate for MdpK, but the production of some chrysophanol 2 can still be observed, implying that some reduction may occur spontaneously.

Simpson and colleagues proposed, by extension, that in aflatoxin biosynthesis AflX actually reduces versicolorin A (VA) 51 to its dihydroquinone, before the action of the hydroquinone reductase AflM (Scheme 4). In this proposal, the hydroxyketone product of AflM 52 would then undergo dehydration and spontaneous autoxidation to 6-deoxy-VA 53 in a manner that is analogous with the dehydration and autoxidation leading to chrysophanol 2. However, this would be somewhat inconsistent with the work of Henry and Townsend, which showed that 6-deoxy-VA 53 was not incorporated into aflatoxins, even though versicolorin A 51 was efficiently utilised under similar conditions.^64^ One possibility is that in aflatoxin biosynthesis, AflM catalyses the hydroquinone reduction seen in Scheme 4, but rather than immediately undergoing dehydration, the resulting hydroxyketone 52 is derivatised in a different manner, with dehydration occurring at a later stage. Consistent with this is the fact that although the aflatoxin BGCs include an MdpC-like anthrol reductase (namely AflM), they do not contain an MdpB-like dehydratase.

If AgnL4-like reductases are anthraquinone reductases involved in the conversion of emodin 1 to chrysophanol 2, one obvious question was why homologues are encoded by the geodin 19, trypacidin 21 and pestheic acid 22 BGCs, when these pathways do not require a C-6 deoxygenation. An answer to this question has recently been provided by Lu and colleagues,^75^ who have shown that reduction of an anthraquinone to a hydroquinone is also required prior to the ring cleavage seen in many pathways (see Section 3.1). It therefore appears that these enzymes act as broad anthraquinone reductases, which are involved in the deoxygenation of emodin in relevant pathways, but also the reduction of the anthraquinones such as questin and chrysophanol prior to ring cleavage.

To summarise, the deoxygenation of emodin 1 to chrysophanol 2 begins with reduction of the anthraquinone to a hydroquinone, catalysed by an Agn4/MdpK-like enzyme, followed by a phenol reduction (AgnL6/MdpC), dehydration by a scytalone dehydratase-like enzyme (AgnL8/MdpB) and spontaneous autoxidation to give chrysophanol 2.

## 3 Specific chemistries and their genes across the sub-families

**Fig. 5:**
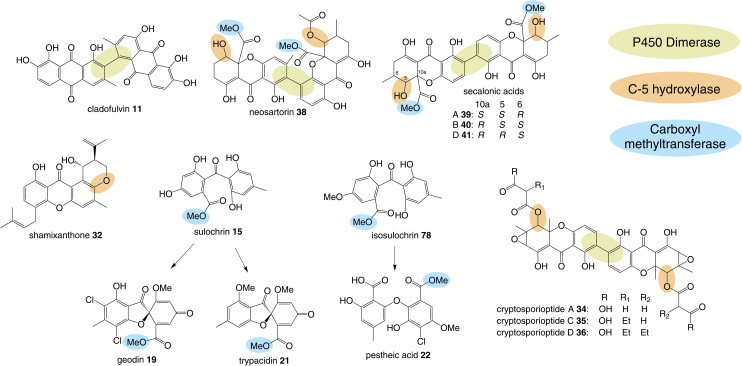
Shared biosynthetic features observed in the emodin-related family of compounds include dimerisation, C-5 hydroxylation and methylation of the carboxyl group.

In addition to being derived from the same early intermediates emodin and chrysophanol, many of the compounds in this family have shared structural features (Fig. 5) that come about through common chemistries in their biosynthetic pathways, and this is reflected by the presence of homologous genes in the biosynthetic gene clusters (BGCs). Some of these will be discussed in this section in anticipation of their role among the specific groups of emodin-derived compounds.

### 3.1 Bienzyme-catalysed ring cleavage

Perhaps the most important shared chemistry in this family of compounds is the oxygenase-catalysed ring cleavage, which cleaves the central ring of anthraquinones to produce a benzophenone. This ring cleavage paves the way for the production of diverse structural classes such as xanthones, grisandienes and the related diphenyl ethers and spiroketals. The only pathways from this family that do not require the action of such an oxygenase are those which retain the anthraquinone core (discussed in Section 4.1).

In the case of the grisandienes, the anthraquinone questin 9 was shown to be converted to the well-known benzophenone sulochrin 15 by *Penicillium glabrum* (previously *P. frequentans*) in 1969^13^ and *A. terreus* soon after in 1970.^76^ In a key study by Fujii and co-workers in 1988,^17^ enzyme activity was detected in a cell free extract of *A. terreus* that converted questin 9 to the intermediate desmethylsulochrin 16. This activity was shown to require both NADPH and molecular oxygen and thus the enzyme – named questin oxygenase – was classified as an oxygenase and proposed to catalyse a Baeyer–Villiger type oxidation. The activity of the extract was lost upon fractionation, but restored when two of the fractions were combined, implying that ‘questin oxygenase’ is actually a complex of enzymes, and authors proposed that additional factors such as electron transfer proteins may be required.

**Fig. 6:**
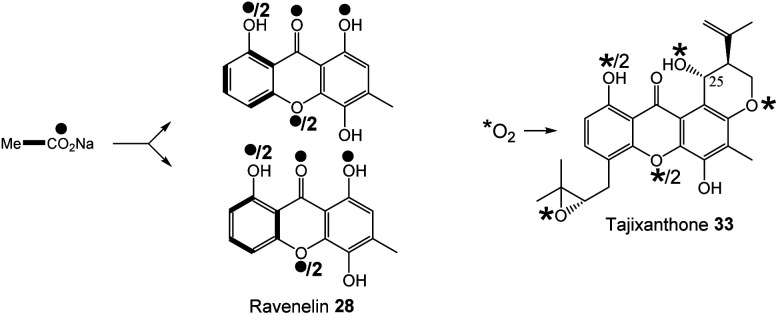
Incorporation patterns of labelled acetates and oxygen into ravenelin 28^77^ and tajixanthone – 33.^61^

Early indications for such a cleavage in xanthone biosynthesis came from feeding studies with [1,2-^13^C_2_]-acetate to the ravenelin 28 producer, *Helminthosporium ravenelii*. Randomisation of intact acetate units in ring A (Fig. 6) indicated that biosynthesis of the xanthone was likely to proceed *via* a benzophenone intermediate with a symmetrical aromatic ring such as monodictyphenone 12.^77^ Evidence for an oxidative process was then obtained by Vederas and coworkers,^78^ who used feeding experiments to incorporate doubly labelled [1-^13^C, ^18^O_2_]-acetate into ravenelin 28, the results of which were consistent with an oxidation removing the 10-carbon of emodin 1 and introducing an atmospheric oxygen atom that was randomised to 8/10a (anthraquinone numbering), prior to ring closure to the xanthone. Subsequent studies on tajixanthone 33 using ^18^O_2_, provided definitive evidence for the oxidative origin of the equivalent oxygens in the xanthone core (Fig. 6).^61^

Upon identification of the geodin 19 (*ged*) BGC, Mortensen and colleagues used bioinformatic analyses to infer that *gedK* could encode the required questin oxygenase.^52^ Consistent with this is the presence of homologous genes in all clusters requiring ring cleavage (see Table 1), and as discussed previously in Section 2.2, deletion of these genes has consistently led to accumulation of anthraquinones and a loss of later compounds.

**Scheme 5:**
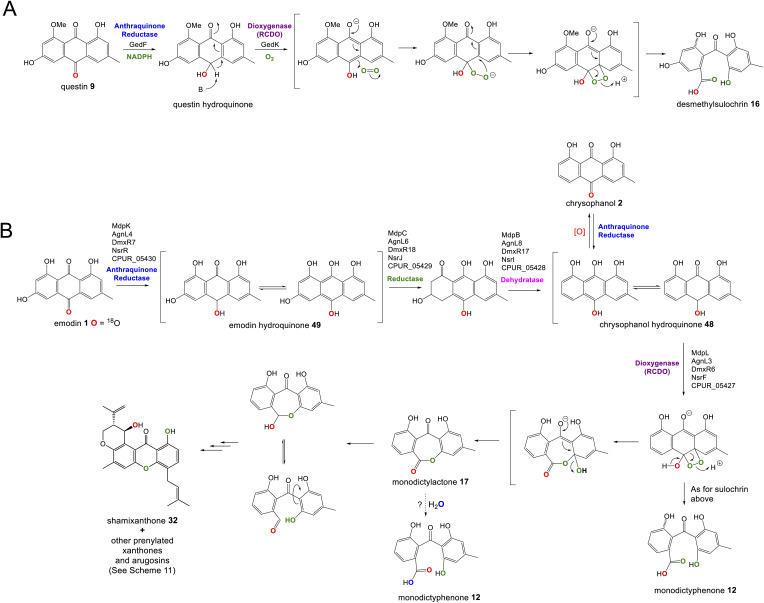
Pathways from anthraquinones to benzophenones. (A) The conversion of questin 9 to desmethylsulochrin 16 in the geodin biosynthetic pathway, showing the likely mechanism of the cofactor free dioxygenase GedK. Based on the proposal by Lu and colleagues.^75^ (B) The likely route from emodin 1 to chrysophanol 2, monodictyphenone 12 and the later prenylated xanthones in *Aspergillus nidulans*.

Recent seminal work^75^ on the geodin pathway has further confirmed that the ring cleavage from an anthraquinone to a benzophenone is oxidative and that as originally proposed by Fujii and coworkers,^17^ it does require multiple enzymes. However, it does not involve a Baeyer–Villiger monooxygenase (BVMO) – a long held belief – but rather a reductase and dioxygenase. Through a combination of *in vivo* gene disruptions, *in vitro* enzymatic analysis, and ^18^O_2_ chasing experiments, Lu and colleagues demonstrated that ring cleavage from questin 9 to desmethylsulochrin 16 proceeds *via* the reduction of 9 to questin hydroquinone, followed by cleavage of C-10/C-4a bond to give desmethylsulochrin 16 (Scheme 5A).^75^ As indicated, both oxygen atoms from the labelled O_2_ molecule introduced into the key dioxetane intermediate by the, now termed, ring-cleaving dioxygenase (RCDO) are retained in the proposed rearrangement to give desmethylsulochrin. The initial reduction of the C-10 keto group is catalysed by the NADPH dependant reductase GedF, with cleavage of questin hydroquinone then being catalysed by GedK–an unusual cofactor free dioxygenase. As mentioned in Section 2.2, these findings answer the riddle as to why a pathway that does not involve deoxygenation of emodin would require a homologue to the anthraquinone reductase AgnL4. It has also led to an updated proposal for the pathway from emodin 1 to monodictyphenone 12, based on the likely functional homology of all ring-cleaving dioxygenases (RCDOs) in this family. In this proposal, chrysophanol hydroquinone 48 is the substrate for ring cleavage to produce monodictyphenone. We know that 48 is an intermediate in the deoxygenation of emodin 1 to chrysophanol 2 (see Section 2.2), so it is possible that 48 is fed directly to the dioxygenase (Scheme 5B). However, it is also likely that 48 is spontaneously oxidised to chrysophanol and that the anthraquinone reductases present in these pathways plays a role maintaining the presence of the reduced 48 (Scheme 5B). This fits with the fact that feeding studies have shown chrysophanol to be an intermediate, being incorporated into the prenylated xanthones (see Section 2.2). Thus, it seems that reduction of an anthraquinone to a hydroquinone is a key feature of these related biosynthetic pathways, and that in many cases these reductases are likely to be multifunctional, acting at different stages of the pathway, on different anthraquinones. The biosynthetic pathway for cladofulvin is another such example (see Section 4.1 and Scheme 7).

While unequivocally demonstrating the involvement of RCDOs rather BVMOs in these crucial AQ ring cleavage processes, the specific details may differ depending on the specific substrate and RCDO. Previous labelling work on shamixanthone 32 biosynthesis in *Aspergillus variecolor* using a judicious mixture of ^18^O_2_ and ^16^O_2_ to differentiate between possible monooxygense or dioxygenase mechanisms for ring cleavage,^61^ had been interpreted in favour of two monooxygenases events being involved. Retention of a full oxygen 18 at C25 of shamixanthone 32 ruled out the intermediacy of a carboxylic acid such as monodictyphenone and favoured a lactone, monodictylactone 17 - a previously hypothetical intermediate that has now been isolated as a natural product from the agnestin producer, *Paecilomyces variotii*.^34^ Both of the above observations can be reconciled by the slightly different ring opening of the dioxygenase derived dioxetane intermediates shown in Scheme 5B. Formation of the lactone and subsequent dehydration to form the xanthone rationalises the loss of one dioxygenase derived oxygen label. Monodictyphenone 12 may also be derived by hydrolysis of monodictylactone 17 rather than directly from the oxetane (Scheme 5B). Indeed, the majority of metabolites featuring a partially (*e.g.* neosartorin 38) or fully (*e.g.* cryptosprioptide 34) reduced anthraquinonoid carbonyl may well be derived *via* monodictylactone 17.

### 3.1 Dimerisation

**Scheme 6:**
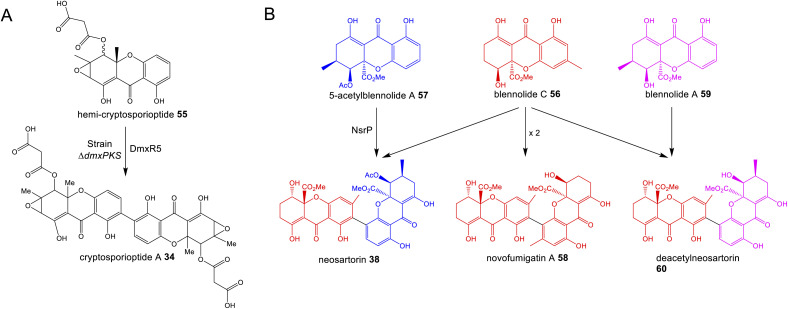
(A) Feeding hemi-cryptosporioptide 55 to the *dmxPKS* mutant strain, restores production of cryptosporioptide (A), demonstrating that the DmxR5 catalysed dimerisation is the last step of the pathway.^32^ (B) Alternate couplings of blennolide monomers by NsrP.^36^

A feature that is common to multiple pathways is oxidative coupling to produce the many dimeric compounds in this family, including cladofulvin 11, the secalonic acids (*e.g.*39–41), cryptosporioptides (*e.g.*34–36) and neosartorin 38. In 2016 the C-2 – C-4′ dimerase from the cladofulvin pathway was identified as the P450 monooxygenase ClaM, which catalyses the oxidative dimerisation of two molecules of the anthraquinone nataloe-emodin 54^31^ (see Section 4.1 for more details). Greco and colleagues then identified a homologous P450, DmxR5 as being encoded by the BGC for the cryptosporioptides and confirmed through gene deletions that this enzyme catalyses the C-2 – C-2′ dimerisation of xanthone monomers to produce the dimeric final compounds.^32^ Feeding the monomeric compound, hemi-cryptosporioptide 55 – produced by the Δ*dmxR5* strain – to a cryptosporioptide PKS mutant (Δ*dmxPKS*) confirmed for the first time that xanthone formation (see Section 4.2.1) precedes dimerisation (Scheme 6A).^32^ A comparative bioinformatics analysis undertaken during the investigation into cryptosporioptide biosynthesis also identified the homologous P450 dimerase encoding genes in the secalonic acid BGCs, although these were outside the originally annotated secalonic acid cluster of *Claviceps purpurea*.^32^ Recent work on the secalonic acid cluster from *Aspergillus aculeatus*^79^ has now confirmed the identity of the P450 AacuE as a dimerase and has shown *via* feeding studies that it can accept diverse monomers to produce a range of homo- and heterodimeric xanthones, including those not naturally produced by *A. aculeatus*. Despite the relaxed substrate specificity, AacuE does demonstrate high regioselectivity and stereoselectivity, with all the resulting dimers having 2−2′ linkage and *S*a axial chirality.^79^ The heterodimer neosartorin 38 is produced *via* the oxidative C-2 – C-4′ coupling of blennolide C 56 and 5-acetylblennolide A 57, and again this is catalysed by a P450, NsrP.^36^ NsrP is an intriguing dimerase in that is shows a clear preference for dimerising the two distinct monomers, with neosartorin being the only major xanthone (hetero)dimer produced by wild-type *Aspergillus novofumigatus*. Upon deletion of the *O*-acetyltransferase NsrL, which abolishes production of the monomer acetylblennolide A 57, NsrP is capable of coupling two molecules of blennolide C 56 to produce the homodimer novofumigatin A 58 or coupling blennolide C 56 with blennolide A 59 to produce deacetylneosartorin 60, showing that although NsrP demonstrates a strong substrate preference, it is capable of utilising at least three different xanthone monomers as substrates (Scheme 6B).^36^ All couplings are non-symmetrical between C-4′ of blennolide C 56 and C-2 of either blennolide A 59, 5-acetylblennolide A 57 or blennolide C 56.

### 3.2 C-5 hydroxylation

Another chemistry present in multiple pathways is C-5 hydroxylation (Fig. 5). Gene deletion studies identified the monooxygenase MdpD as the likely C-5 hydroxylase involved in the biosynthesis of the prenylated xanthones (*e.g.* shamixanthone 32 and tajixanthone 33) produced *inter alia* by *Aspergillus nidulans*.^63^ Based on homology to MdpD, DmxR9 from the cryptosporioptide biosynthetic pathway was proposed as catalysing the C-5 hydroxylation required in that pathway, and by the same logic, CPUR_05423 was identified as the likely C-5 hydroxylase involved in secalonic acid biosynthesis, although these roles have not been experimentally confirmed.^32^ A further homologue, NsrK, was identified through gene knock-outs as installing the C-5 hydroxyl group in neosartorin 38 biosynthesis. This was initially proposed to proceed *via* an epoxidation,^36^ but later *in vitro* work, using the epoxide trapping reagent *N*,*N*-diethyldithiocarbamate (DTC) suggests that this is not the case.^80^ The biosynthesis of neosartorin 38 involves a branching pathway where the RCDO cleaves either the C-10/C-10a bond or the C-10/C-4a bond of chrysophanol hydroquinone 2 (see Scheme 8). Subsequent enzymes, including the C-5 hydroxylase NsrK, are therefore capable of functioning in either the presence or absence of a methyl group on the aromatic ring.

### 3.3 C-12 methyl esterification

Multiple sub-groups feature a distinctive C-12 methyl ester (secalonic acid numbering), generated *via* methylation of the carboxyl group that results from the AQ precursor C-10 ketone during dioxygenase-catalysed ring cleavage. In the trypacidin 21 pathway, the carboxyl methyltransferase can be putatively identified through comparisons with the geodin 19 pathway and a process of elimination. Both the geodin 19 and trypacidin 21 pathways involve two homologous methylations, emodin *O*-methylation to give questin 9, and methylation of the carboxyl group of desmethylsulochrin 16 to give sulochrin 15. In line with this, there are two homologous pairs of *O*-methyltransferases encoded by the two gene clusters: TpcA/GedA and TpcM/GedG. Deletion of *tpcA* led to an accumulation of emodin 1, thus identifying TpcA/GedA as the emodin *O*-methyltransferase and by extension TpcM/GedG as the likely carboxyl *O*-methyltransferases. This is consistent with the presence of *tpcM*/*gedG* homologues in the BGCs of all other xanthones that have a methyl ester, namely the secalonic acids (*e.g.*39-41) and neosartorin 38 (See Fig. 5 and Table 1). The neosartorin carboxyl *O*-methyltransferase NsrG was subject to gene-deletion studies, but although neosartorin 38 production was abolished, unfortunately no related metabolites accumulated.^36^ However, the isolation of the methyl ester moniliphenone 61 and its monodictyphenone analogue 63 (2,2′,6′-trihydroxy-4-methyl-6-methoxy-acyl-diphenylmethanone) (see Scheme 9) from *Aspergillus novofumigatus*, led to the conclusion that NsrG catalyses the methylation of both monodictyphenone 12 and cephalanone F 14 on the branching pathway of neosartorin 38 biosynthesis.^36^

## 4 Branched pathways to ‘sub-families’

### 4.1 The wider anthraquinone family

Although the anthraquinones emodin 1 and chrysophanol 2 are discussed here primarily as key intermediates in the pathways to compounds such as grisandienes and xanthones, fungal anthraquinones themselves represent a large and diverse group of secondary metabolites.^22^

Various anthraquinone compounds that retain the carboxyl group are likely to be derived from endocrocin 5. In the secalonic acid producer *C. purpurea*, endocrocin 5 appears to be hydroxylated at C-4 to produce an anthraquinone called clavorubin 6 (see Scheme 2). Although the enzyme responsible for this step has not been confirmed, two candidates; CPUR_05423 and CPUR_05431, were suggested based on a bioinformatic analysis of the ergochrome BGC.^35^ Comparative bioinformatics undertaken during investigations into the cryptosporioptide gene cluster^32^ identified CPUR_05432 as the likely C-5 hydroxylase required for secalonic acid biosynthesis (see Section 3.2; Fig. 5),^32^ and as such, the unique monooxygenase CPUR_05431 may now be considered the most likely candidate for the hydroxylation in the biosynthesis of clavorubin 6.

In various *Dermocybe* fungi, *O*-methylation and hydroxylation of endocrocin 5 likely lead to the production of dermolutein 7 and dermorubin 8 (Scheme 2), although it must be noted that the biosynthesis of anthraquinones is largely unexplored in basidiomycetes, and the genes involved in the production of these compounds have not been identified.

In addition to containing the core anthraquinone producing genes (PKS, MβL-TE and anthrone oxidase), the endocrocin 5 gene cluster of *A. fumigatus* also contains a 2-oxoglutarate-Fe(ii)-type oxidoreductase; *encD*. Disruption of *encD* led to a 6-fold increase in endocrocin 5 yields, suggesting that endocrocin is the substrate for EncD, which may catalyse the conversion of 5 to an as-yet unknown compound.^26^

Anthraquinones derived from emodin 1 include its 7-hydroxylated derivative, nataloe emodin 54 and its dimer, cladofulvin 11, as well as islandicin 3, (5-hydroxy-chrysophanol). Although islandicin 3 may appear to be an obvious intermediate in many emodin-related pathways, feeding studies have not supported this theory, seeing no incorporation into shamixanthone 32 (unpublished) and very little incorporation into the secalonic acids.^58^ Townsend proposed that islandicin 3 is a shunt metabolite from an oxidised chrysophanol intermediate prior to ring cleavage.^64^

**Scheme 7:**
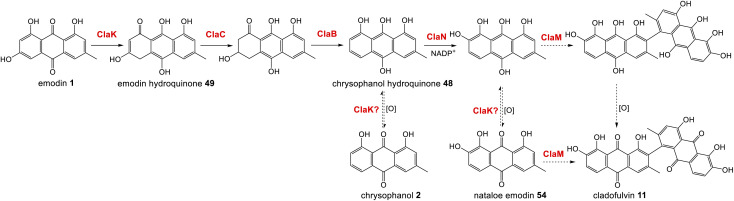
The proposed route from emodin 1 to the dimeric anthraquinone cladofulvin - 11. It is currently unknown whether nataloe emodin 54, or the reduced form, are the substrate for ClaM, although feeding studies^31^ suggest the latter. It is quite possible that the anthraquinone reductase ClaK functions to reduce emodin, chrysophanol and nataloe emodin to the corresponding hydroquinones, to generate the correct substrates for the relevant enzymes. The assignment of ClaK and ClaN is based on comparative bioinformatics conducted during the writing of this review. The roles of ClaC, ClaB and ClaM have previously been experimentally confirmed.^31^

The gene cluster for cladofulvin 11 was published in 2016 and has been fairly thoroughly characterised through gene disruptions and heterologous expression.^31^ As discussed previously, the expression of genes from the *cla* BGC in *A. oryzae* has contributed to our understanding of emodin 1 and chrysophanol 2 biosynthesis, and the cytochrome P450 ClaM was the first dimerase from this biosynthetic family to be experimentally confirmed.^31^ Disruption of ClaM led to a loss of cladofulvin 11, and a clear accumulation of both emodin 1 and nataloe emodin 54. This identified nataloe emodin 54 as the likely immediate precursor to cladofulvin 11, although feeding 54 to a ClaM expressing *A. oryzae* strain did not lead to cladofulvin 11 production, highlighting the possibility that the reduced form of nataloe emodin, may be the true substrate for ClaM (Scheme 7).^31^

The only enzymes not previously attributed to specific steps in the cladofulvin pathway are the two oxidoreductases; ClaK and ClaN. One or both were assumed to be involved in the production of nataloe emodin 54 from chrysophanol 2, but co-expression of both ClaK and ClaN in *A. oryzae* failed to yield any new products when fed with chrysophanol 2. Collemare and co-workers suggested therefore, that chrysophanol hydroquinone 48, not chrysophanol 2 itself, may be the intermediate in the pathway (Scheme 7). This is consistent with the pathway from emodin 1 to chrysophanol 2 proceeding *via* emodin hydroquinone 49 and chrysophanol hydroquinone 48 and is also consistent with the lack of 2 detected in mutant strains such as Δ*claM* strains, even though an accumulation of earlier intermediates such as emodin 1 is observed. As to which enzyme is involved in the required C2 hydroxylation to produce 54, comparative bioinformatics now allows us to rule out ClaK as a likely candidate. ClaK is a clear homologue to the anthraquinone reductase AgnL4 from the agnestin pathway (53.8% protein identity), which has been proposed to catalyse the required reduction of emodin 1 to emodin hydroquinone 49 (See Section 2.2). The apparent requirement for hydroquinones as substrates for multiple enzymes in the cladofulvin pathway highlights the possibility that ClaK acts at multiple points, producing and/or maintaining the reduced compounds (Scheme 7).

This putative assignment for ClaK leaves ClaN as the likely candidate for the hydroxylation required to produce nataloe emodin 54 and cladofulvin 11 (Scheme 7).

### 4.2 Xanthones

Fungal xanthones are a large and complex family of compounds, which demonstrate significant chemical diversity and exhibit such a wide array of bioactivities that they have earned themselves the status of ‘privileged structures’. The details of individual biosynthetic pathways are too diverse to discuss fully, but rather, specific shared characteristics and the types of enzymes involved in xanthone biosynthesis will be summarised to give an overview of their biosynthesis.

**Scheme 8:**
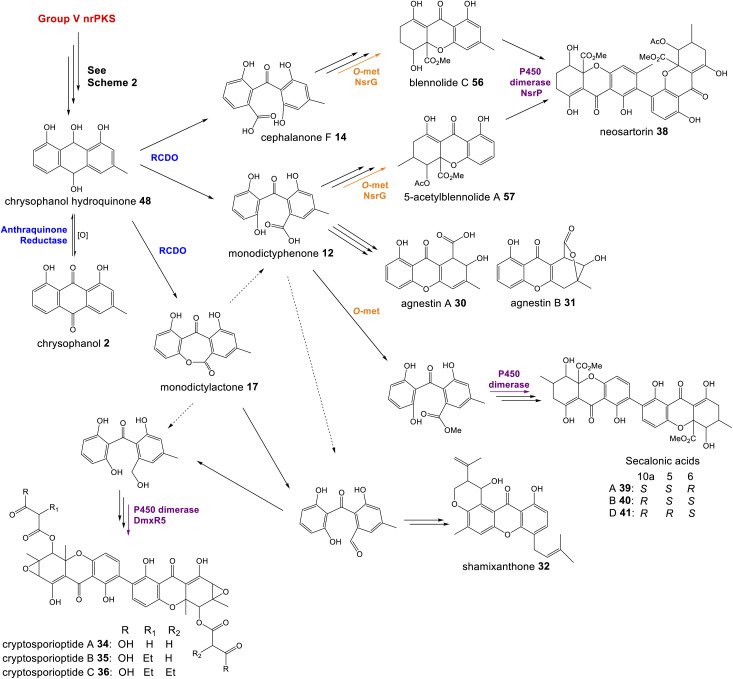
The oxidative ring cleavage of chrysophanol hydroquinone, various tailoring steps and xanthone formation generates a diverse range of final compounds.

As discussed in Section 3.1, the first step in the transition from an anthraquinone core to the xanthone core is most likely a dioxygenase-catalysed cleavage. There are examples of fungal xanthones that retain the C-6 hydroxyl group of emodin 1, such as those recently isolated from *Bipolaris sorokiniana.*^81^ In such cases it is likely that emodin 1, or its corresponding hydroquinone 49, is the substrate for oxidative cleavage. However, in the majority of studied fungal xanthone pathways, including all those discussed here, deoxygenation of emodin 1 occurs prior to the ring cleavage (see Section 2.2). An interesting characteristic of the xanthone ring-cleaving dioxygenases (RCDOs) is their varied regioselectivity. Chrysophanol hydroquinone 48 can be converted to either monodictyphenone 12, or its isomer cephalanone F 14, depending on whether the dioxygenase interacts with the methyl-containing or non-methyl aromatic ring of 48 (Scheme 8). 12 is formed through cleavage of the C-10/C-10a bond whereas 14 is formed through the alternative cleavage of C-10/C-4a bond. Most RCDOs appear to exhibit somewhat relaxed regioselectivity, as demonstrated by the fact that *Graphiopsis chlorocephala*, the species from which cephalanone F 14 was originally isolated, also produces some monodictyphenone 12.^82^ Similarly, *Paecilomyces variotii* – the species that produces the monodictyphenone-derived agnestins (*e.g.*30 and 31) – also produces some cephalanone F 14, albeit as a relatively minor metabolite.^34^ The RCDO from the neosartorin 38 pathway, NsrF, demonstrates very relaxed regioselectivity, interacting with either the methyl or non-methyl-containing aromatic rings equally, thus allowing a branched pathway and production of a heterodimer (Scheme 8).^36^ A recent study, on the secalonic acid pathway from *Aspergillus aculeatus*, has shown that in this system, the relatively strict selectivity of the RCDO AacuH plays a key role in determining the output of the pathway.^79^ When enzymes downstream of the RCDO were expressed in an *A. oryzae* strain capable of producing both benzophenone intermediates (*i.e.* monodictyphenone 12 and cephalanone F 14), a wide range of different blennolides were produced, including many not naturally produced by *A. aculeatus*.^79^

Most xanthone pathways involve a variety of tailoring steps, some of which are common to other emodin-derived pathways. Methylation of the C-12 carboxyl group (equivalent to C-10 of chrysophanol) and C-5 hydroxylation, for example, are seen across this family of compounds and are discussed in Section 3. It is noteworthy that C-12 is found in all possible oxidation states across this family of compounds: – carboxylic acid, lactone (agnestins A 30 and B 31), methyl ester (blennolides, *e.g.*56, 59; secalonic acids, *e.g.*39–41), aldehyde (variecoxanthones, *e.g.*66), primary alcohol (dicerandrols)^83^ and methyl (cryptosporioptides 34–36). Other modifications at C-5 include the *O*-acetylation seen in the neosartorin 38 pathway, which exhibits strict substrate specificity – occurring on only one of the monomers; blennolide A 59.^36^ The cryptosporioptides are particularly complex and highly decorated xanthones, as is reflected by the large number of pathway specific genes seen in the *dmx* cluster (Table 1). They contain an unusual C-10 methyl group, and in the cases of cryptosporioptides B 35 and C 36, an unusual ethylmalonate subunit attached to the C-5 hydroxyl.^32^ Using a series of gene knock-outs Greco *et al.* determined that both an hrPKS (DmxL2) and acyl CoA carboxylase (DmxL1) are required for the ethylmalonate subunit.^84^ They proposed that dmxL2 produces butyrate, which is carboxylated (DmxL1) to form ethylmalonate, before being esterified to *O*-5 by DmxR13.

#### 4.2.1 Formation of the xanthone core

There have been many speculations regarding the formation of the xanthone structure itself, and whether such cyclisation occurs spontaneously (*e.g.* norlicheoxanthone is known to form spontaneously from dehydration of a benzophenone^85^ - see Box 2 in Section 4.3.2) or whether specific enzymes are involved. The exact mechanism no doubt varies depending on the exact pathway and the type of xanthone, *i.e*. fully aromatic (*e.g.* shamixanthone 32), dihydroxanthone (*e.g.* agnestins 30 and 31) or tetrahydroxanthone (*e.g.* secalonic acids; cryptosporioptides: *e.g.*34–36; and neosartorin 38). In 2016, during investigations into cryptosporioptide biosynthesis, Greco *et al.* speculated that small snoAL-like enzymes may act as xanthone cyclases.^32^ DmxR10 was identified as a potential xanthone cyclase based on the presence of homologues in all identified xanthone clusters and homology modelling using Phyre2, which modelled DmxR10 onto Trt14 from *Aspergillus terreus* with complete confidence. Trt14 is involved in an interesting ring formation in terretonin biosynthesis,^86^ where it catalyses multistep, sequential ester bond cleavage and formation to reconstruct the D-ring.^86^

**Scheme 9:**
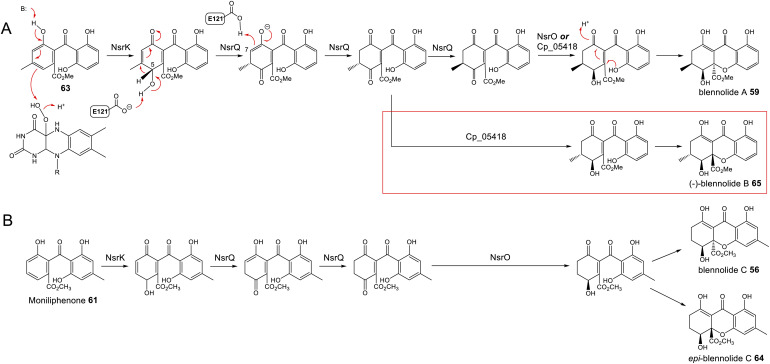
Later steps in the biosynthesis of blennolides A 59 (Panel A) and C 56 (Panel B), from their methyl ester precursors, 63 and 61 respectively. These pathways were proposed by Wei & Matsuda^80^ based on *in vitro* assays with NsrK, NsrQ and NsrO. Replacing NsrO in the blennolide A *in vitro* assay (panel A) with the homologous ketoreductase from *C. purpurea* (Cp_05418) led to the additional generation of (−)-blennolide B 65 (red box). The authors recognised that the C-5 ketoreduction could occur either prior to, or after, cyclisation to give the xanthone core.^80^

To address the mechanism behind tetrahydroxanthone formation, Wei and Matsuda^80^ used both *in vivo* and *in vitro* experiments to reconstitute the biosynthesis of the neosartorin xanthone monomers: blennolides A 59 and C 56. Initial attempts to express the 11 genes proposed to be involved in blennolide biosynthesis led to the production of benzophenones rather than xanthones, indicating that a gene required for xanthone formation was missing. Based on the previous identification of DmxR10 as a potential xanthone cyclase, two homologues from the *nsr* cluster, NsrQ and NsrS, were then also expressed in *A. oryzae* and this identified NsrQ as the missing link in xanthone biosynthesis. Addition of NsrQ to the heterologous expression experiments allowed double bond reduction and cyclisation to the xanthone. To fully elucidate the order of events and mechanisms, *in vitro* work was undertaken. Due to unstable intermediates, *in vitro* assays were carried out using a combination of the three enzymes predicted to be involved in the later stages of blennolide biosynthesis: NsrK (C-5 hydroxylase), NsrQ (xanthone cyclase/isomerase) and NsrO (C-5*S* Reductase). The K/Q/O enzyme combination was fed with either one of the two predicted methyl ester precursors: moniliphenone 61, and its isomer 63 – the products of NsrG methylation of cephalanone F 14 or monodictyphenone 12 respectively. Moniliphenone 61 was converted into blennolide C 56 as well its C-10a epimer *epi*-blennolide C 64. 63 was converted into blennolide A 59 (Scheme 9).^80^

Various *in vitro* assays were carried out,^80^ including those conducted in D_2_O, or containing (4S)-[4-^2^H]NADPH to identify NADPH-dependant reductions, and this allowed a pathway to be proposed for the biosynthesis of the blennolides (Scheme 9). Starting the with methyl ester precursors 63 (Scheme 9A) or 61 (Scheme 9B), NsrK installs a hydroxyl group at C-5. This role was previously indicated by gene knock-out experiments^36^ and is consistent with homologues in other clusters requiring C-5 hydroxylation (see Section 3). NsrQ is then thought to catalyse tautomerisations to generate the ketone at C-5, and in the case of blennolide A 59 biosynthesis, this is followed by a methyl group epimerization that inverts the stereochemistry at C-6 (Scheme 9A). The action of NsrQ allows dearomatizing cyclisation to occur, generating the tetrahydroxanthone architecture. NsrO was proven, using deuterium labelling, to be a C-5*S* ketoreductase – which could act either before or after cyclisation and xanthone formation.^80^

Wei and Matsuda began investigating the catalytic function of the xanthone cyclase NsrQ *via* mutational experiments.^80^ Modelling the protein structure of NsrQ using Phyre2 identified the likely substrate binding site, comprising 16 amino acid residues, and mutating each of these identified Glu121 as the probable catalytic residue. The authors proposed that Glu121 is responsible for deprotonation of the C-5 hydroxyl group allowing a 1,2-hydride shift to occur from C-5 to C-6, followed by reprotonation at C-7 (Scheme 9A).

**Scheme 10:**
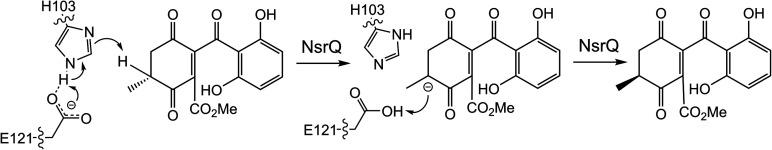
Proposed mechanism for the methyl group epimerisation catalysed by NsrQ in the pathway to blennolide A 59 (see Scheme 9A).^87^

**Fig. 7:**
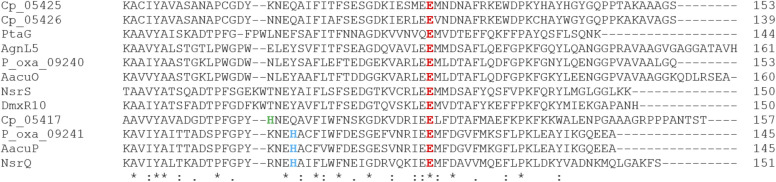
An alignment of putative xanthone cyclase/isomerases shows the conserved catalytic residue, E121, highlighted in red.^80^ Highlighted in blue is the base catalyst histidine thought to be required for the methyl group epimerisation (see Scheme 10),^87^ with a nearby histidine in Cp_05417 highlighted in green.

More recently, a series of comprehensive studies involving site directed mutagenesis and X-ray crystallography, have further uncovered the structural basis for these fascinating multifunctional enzymes.^87^ As predicted, Glu121 was shown to be the critical residue for the initial allylic alcohol isomerisation. For the subsequent methyl group epimerisation, Glu121 as well as His103 were shown to be necessary, with evidence suggesting that His103, activated by Glu121, abstracts the C-6 hydrogen atom to generate a carbanion intermediate and then Glu121 protonates from the opposite side of the molecule (Scheme 10).^87^ An alignment of NsrQ and homologous isomerases from clusters discussed in this review shows that they all contain the conserved glutamine residue, but interestingly only a select few contain the histidine residue considered necessary for the inversion (Fig. 7). The potential implications of this will be discussed further in the following section on xanthone stereochemistry.

#### 4.2.2 Xanthone stereochemistry

**Scheme 11:**
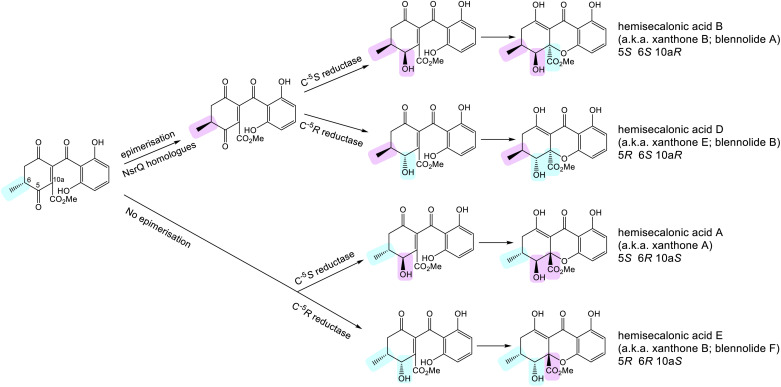
Possible routes to xanthone stereoisomers, starting from the intermediate proposed by Wei and Matsuda.^80^ Whether or not epimerisation of the C-6 methyl group occurs would go on to determine the stereochemistry of the *trans*-invariant C-6:C-10a position. This may be further controlled by the substrate specificity of the C-5 ketoreductase, which may accept one or both C-6 enantiomers. The ketoreductase then sets the stereochemistry at C-5.

Another intriguing aspect of di/tetrahydro-xanthones, which is starting to be understood in biosynthetic terms, is their stereochemistry. The secalonic acids are a perfect study of stereochemistry as they are all stereoisomers; their constituent monomers differing only in the stereochemistry at C-5 or at the *trans*-invariant C-6:C-10a position (Scheme 11). The varied bioactivity of the secalonic acids highlights just how important understanding their stereochemistry is. Secalonic acid A, for example exhibits antitumour and neuroprotective affects, whereas its enantiomer, secalonic acid D, is a highly toxic teratogen. Firstly, to focus on the C-5 position, Greco *at al*. determined that the observed stereochemistry is perfectly consistent with a bioinformatic analysis of likely C-5*R* and C-5*S* ketoreductases encoded by the various biosynthetic gene clusters.^32^*C. purpurea* produces secalonic acids that exclusively have 5*S* stereochemistry, and its cluster encodes one C-5*S* ketoreductase, namely CPUR_05418. *P. oxalicum* exclusively produces secalonic acids with 5*R* stereochemistry and encodes a predicted C-5*R* ketoreductase, namely P_oxa-09231. *A. aculeatus* produces secalonic acids with both 5*S* and 5*R* stereochemistry and contains homologues to both Cp_05418 (AacuD) and P_oxa-09231 (AacuF). Both the cryptosporioptides and neosartorin 38 have C-5*S* stereochemistry and in line with this they both contain C-5*S* ketoreductases with homology to Cp_05418. As mentioned previously, the role of NsrO as a C-5*S* reductase has been demonstrated by Wei and Matsuda through *in vitro* assays with (4S)-[4-^2^H]NADPH.^80^

Control of the C-6/C-10a stereochemistry is perhaps a little more complex and appears to involve both the snoAL-like isomerases and the C-5 ketoreductases. As discussed previously, the snoAL-like protein NsrQ has been proposed to catalyse tautomerisation followed by an epimerisation to invert the stereochemistry of the C-6 methyl group in blennolide A 59 biosynthesis (Scheme 9A).^80,87^ Setting the stereochemistry at C-6 would then later determine the C-10a stereochemistry as heterocyclisation to produce the xanthone core would occur stereoselectively to prevent the 1,3-axial–pseudoaxial interaction between the C-10a methyl ester and the C-6 methyl group. This reasoning is supported by the fact that both *in vivo* and *in vitro* production of blennolide C 56 - where the methyl group is located on the other ring–also produced *epi*-blennolide C 64 (Scheme 9B).

It is plausible that the presence of multiple snoAL-like proteins in the BGCs for secalonic acids aids the production of xanthones with differing C-6/C-10a stereochemistry, either by catalysing both the isomerisation and epimerisation, as seen with NsrQ, or only the initial allylic alcohol isomerisation but no epimerisation. Indeed, both *A. aculeatus* and *P. oxalicum* – two secalonic producing fungi – encode two such enzymes, one containing the histidine residue thought to be required for epimerisation^87^ and one lacking this residue. This is in contrast to the cryptosporioptide and agnestin BGCs, which contain only one homologue per pathway, lacking the histidine residue. *C. purpurea*, another species known to produce secalonic acids with both 6*S* and 6*R* stereochemistry, encodes three snoAL-like proteins and although none contain a histidine that perfectly aligns with His103 of NsrQ, one protein, Cp_05417, does contain a histidine residue just three residues upstream (Fig. 7). As more of these enzymes are characterised in detail, it will become apparent whether the histidine is universally required for such stereochemical inversions, and whether its presence or absence can be used to predict the activity of the enzyme and resulting stereochemical output of the biosynthetic pathway.

Work on the neosartorin pathway by Wei and Matsuda also uncovered an interesting role for the C-5 reductase in controlling the stereochemistry at C-6/C-10a – not through its catalytic activity but through its substrate specificity.^86^ Assays containing the neosartorin 38 ketoreductase NsrO only led to the generation of blennolide A 59. However, if NsrO was replaced with the homologous ketoreductase from *C. purpurea*, Cp_05418, two diastereomers were generated; blennolide A 59 (a.k.a. xanthone B – 10a*R*) and (-)-blennolide B 65 (Scheme 9). They proposed that this was due to the epimerisation at C-6 being required prior to substrate acceptance by NsrO, while Cp_05418 may be capable of accepting either enantiomer – before or after epimerisation. Carrying out the assays with D_2_O led to incorporation of one additional deuterium atom at C-6 in blennolide A 59, but not in (-)-blennolide B 65, which supports the theory that in the pathway to blennolide A 59 an epimerisation occurs. Again, this is consistent with the stereochemistry of secalonic acids produced by *C. purpurea*; although they all demonstrate C-5*S* stereochemistry, *C. purpurea* produces secalonic acids with both 10a*S* (*e.g.* secalonic acid A) and 10a*R* (*e.g.* secalonic acid B) stereochemistry.

In conclusion, it seems that the stereochemistry of xanthones is controlled by two key enzymes in the later stages of xanthone biosynthesis. SnoAL-domain proteins such as NsrQ are thought to catalyse an inversion of stereochemistry at C-6, which later determines the stereochemistry at C-10a. This inversion seems to work in conjunction with the substrate specificity of the downstream C-5 ketoreductases, which appear to have either strict substrate specificity with regards to C-6 stereochemistry (*e.g.* NsrO), or relaxed substrate specificity, accepting both C-6 enantiomers (*e.g.* Cp_05418). The catalytic action of the C-5 reductase then determines the C-5 stereochemistry. In this way the four monomeric units of the secalonic acids could be generated (Scheme 11), different combinations of which then generate the dimeric secalonic acids A – F.

#### 4.2.3 Prenylated xanthones

Ascomycete fungi, particularly *Aspergillus* and *Penicillium* species, have long been known as producers of prenylated xanthones and benzophenones. Early studies involving isotopic labelling determined that prenylated xanthones such as tajixanthone and shamixanthone are derived from anthraquinone intermediates, and chrysophanol in particular.^61,62,88,89^ A series of gene deletions in *A. nidulans* demonstrated that in this species, variecoxanthone 66, emericellin 67, shamixanthone 32 and epishamixanthone 68 are all derived from the monodictyphenone 12 gene cluster (*mdp*), but this cluster lacks genes required for both xanthone formation and prenylation. Further gene disruptions identified two prenyltransferases encoded elsewhere in the genome; XptA and XptB, as being responsible for the required *C*- and *O*- prenylations respectively. Located near *xptB* is a gene named *xptC*, which encodes an oxidoreductase. XptC is thought to catalyse the formation of the dihydropyran ring of shamixanthone 32 and epishamixanthone 68.^63^ Enzymes required for the reduction and cyclisation of a benzophenone intermediate to produce the xanthone core, however, were not identified.

**Scheme 12:**
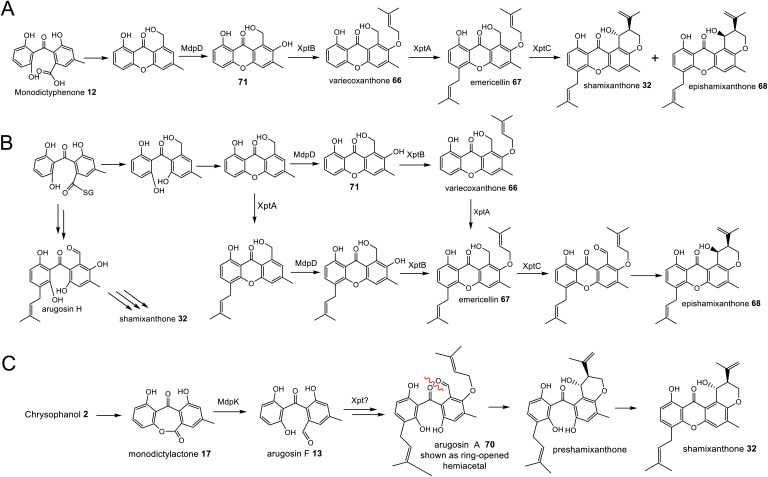
Shamixanthone pathways previously proposed by Sanchez *et al.* (A)^63^ and Simpson (B).^65^ An updated proposal from chrysophanol 2 to shamixanthone 32*via* monodictylactone 17 (C).

Based on the gene disruptions and identification of likely intermediates, Sanchez *et al.*^63^ proposed a simple pathway from monodictyphenone 12 to the prenylated xanthones (Scheme 12A). This was questioned by Simpson in 2012,^65^ who noted various inconsistencies, such as disregarding the incorporation of labelled chrysophanol, and previous findings from fermentations conducted in ^18^O_2_.^61^ In particular, the full retention of a single oxygen atom at C25 ruled out any involvement of carboxylic acid intermediates, *e.g.* monodictyphenone. Also, on the basis of model studies and steric requirements, the plausibility of the aldehyde formed by oxidation of emericellin 67 cyclising to give a dihydropyran with *trans* stereochemistry – as would be required to produce shamixanthone 32 – was questioned. An alternative branching pathway was proposed^65^ which was compatible with previous biosynthetic and labelling studies, and which reconciled the biosynthesis of both the arugosins – prenylated benzophenones produced by *A. nidulans* – and the prenylated xanthones.^65^ In this pathway, chrysophanol 2 was converted *via* mondictylactone 17 to a thiolester intermediate, which served as a branch point for two diverging pathways. Epishamixanthone 68 would be produced from a benzyl alcohol *via* variecoxanthone 66, whereas shamixanthone 32 would be produced *via* arugosin F 13 and the prenylated arugosins such as arugosins A/B 70 (Scheme 12B). Mechanistic *in vitro* studies showed *inter alia* that the aldehyde derived from variecoxanthone underwent Prins cyclisation to give only the *cis* isomer corresponding to epishamixanthone.^61^

The formation of the thiolester was proposed to be mediated by MdpJ, but re-examination of the earlier mutation studies^63^ clearly demonstrate that if involved at all, MdpJ must act much earlier, prior to ring cleavage. It is more likely that the lactone is reduced directly to the lactol, which would be in equilibrium with the corresponding hydroxy-aldehyde arugosin F 13 that would be prenylated to arugosin A 70.

Although an elegant resolution, certain aspects of this pathway have been questioned by Pockrandt *et al.*,^90^ who conducted biochemical characterisation of the *O*-prenyltransferase XptB through *in vitro* assays. XptB was shown to accept and prenylate 1,7-dihydroxy-6-methyl-8-hydroxymethylxanthone 71 to give variecoxanthone 66 – supporting the epishamixanthone 68 branch of the pathway – but did not prenylate any of a number of benzophenones, including arugosin H, as proposed in the pathway to shamixanthone 32 (Scheme 12B). As the prenylated arugosins are co-metabolites in most prenyl-xanthone producers Pockrandt *et al.*^90^ raised the necessity that the pathway to the prenylated benzophenones must involve the action of a prenyltransferase other than XptB, and observed that the genome of *A. nidulans* contains seven genes encoding DMATS, many of which have not been the subject of biochemical characterisation.

A modified pathway, Scheme 12C, in which the crucial dihydropyran cyclisation takes place on a benzophenone rather than the xanthone intermediate, would seem to satisfy the observed results. In support of Scheme 12C, a compound, preshamixanthone, in which dihydropyran ring formation has occurred before xanthone formation, has been isolated from a continuous culture of *Aspergillus nidulans* grown in a chemostat.^91^

### 4.3 Grisandienes and related structural classes

#### 4.3.1 Grisandienes

Two of the best known and most intensively studied grisandienes are geodin 19 and trypacidin 21; toxic grisandienes produced by *Aspergillus terreus* and *Aspergillus fumigatus*, respectively. Grisandiene studies were amongst some of the earliest on this family of compounds, with geodin 19 being shown to be derived from emodin 1 through feeding studies using [UL-^14^C] emodin, published in 1975.^14^

**Scheme 13:**
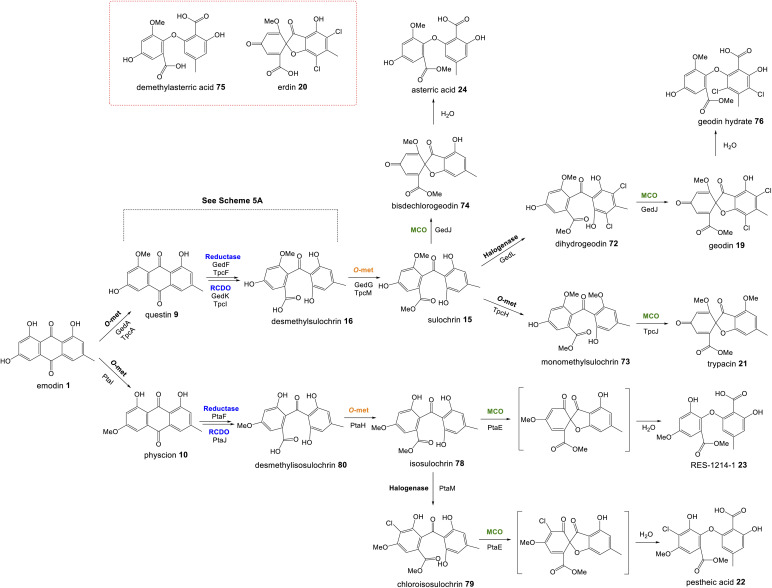
The pathways from emodin 1 to the grisandienes (*i.e.* geodin 19 and trypacidin – 21) and the diphenyl ethers (*i.e.* pestheic acid 22, RES-1214-1 23 and asterric acid −24). Erdin 20 and demethylasterric acid 75 (in red box) have been isolated from the geodin producer *Aspergillus terreus*^96^ and are likely derived from the same biosynthetic pathway.RCDO = ring cleaving dioxygenase. *O*-met = *O*-methyltransferase. MCO = multi-copper oxidase.

The enzymatic steps between emodin 1 and geodin 19 (Scheme 13) were largely delineated by Fujii and colleagues in the 1980s and 1990s, through the detection of enzyme activities in cell-free extracts, followed by protein purification and characterisation. In 1982 the partial purification and characterisation of emodin *O*-methyltransferase (EOM) confirmed that the first step in the biosynthesis of geodin 19 from emodin 1 is *O*-methylation of 1 to questin 9.^15^ Subsequent purification and characterisation of EOM identified it as a SAM-dependant methyltransferase of approximately 53.6 kDa, with strict substrate specificity and which functions as a homohexamer.^19^ As discussed in Section 3.1, questin 9 was then shown to be converted to the benzophenone desmethylsulochrin 16 by cell free extracts of *A. terreus*, with the theoretical enzyme responsible being named questin oxygenase.^17^ We now know that ‘questin oxygenase’ actually comprises two enzymes, a reductase and a dioxygenase, which catalyse the reduction of questin to questin hydroquinone, followed by oxidative ring-cleavage.^75^

The last step of the geodin 19 pathway was similarly identified through enzyme purification and characterisation. An enzyme named dihydrogeodin oxidase (DHGO) was purified and shown to stereoselectively convert dihydrogeodin 72 to geodin 19, catalysing the key ring closure that converts the benzophenone to the closed spiran ring of the grisandienes.^16,92^ The molecular weight of the active enzyme compared to that detected by denaturing SDS-PAGE (153 kDa *vs.* 76 kDa) suggested that DHGO functions as a dimer, and the intense blue colour of the enzyme, with an absorption maxima at 280 and 600 nm, implied that DHGO is a blue copper protein. In 1995 Huang *et al.* reported the successful cloning, sequencing and heterologous expression of the gene encoding DHGO,^93^ representing the first full sequence of a gene involved in geodin 19 biosynthesis, and indeed the whole emodin-related family of compounds. The protein sequence of DHGO showed homology to other known multicopper proteins and was shown to contain four copper binding domains. Heterologous expression in *A. nidulans* and isolation of an active enzyme confirmed the functional identity of the gene.^93^

After a genome sequence became available, the full geodin BGC could be identified (Fig. 2, Table 1). A bioinformatic analysis of the cluster was reported in 2012,^94^ and this was experimentally confirmed through heterologous expression of the full cluster in *Aspergillus nidulans* in 2013, as well as certain gene disruptions.^27^ Disruption of either the PKS GedC, or the regulator GedR – which shares homology with the well-known regulator from the aflatoxin pathway AflR – abolished geodin 19 production. Disruption of a gene named *gedL* identified it as encoding sulochrin halogenase, which catalyses the double chlorination of sulochrin 15 to give dihydrogeodin.^27^

The same paper that reported the heterologous production of geodin 19,^27^ identified a highly homologous gene cluster in the genomes of *A. fumigatus* and *A. fischerianus*, which was proposed to be the trypacidin 21 BGC (Fig. 2, Table 1). Both Mattern *et al.*^28^ and Throckmorton *et al.*^29^ successfully disrupted the proposed trypacidin PKS gene (*tpcC*) in *A. fumigatus*, abolishing the biosynthesis of trypadicin 21 and thus confirming the identity of the BGC. Mattern *et al.* used the PKS mutant strain to investigate the function of 21 in the opportunistic pathogen *A. fumigatus* and proposed that it plays a protective role against phagocytes both in the environment and during the infection process. Throckmorton *et al.* conducted further gene disruptions, including the thioesterase *tpcB* and the regulatory genes *tpcD* and *tpcE*, all of which also abolished trypacidin 21 biosynthesis. Finally, disruption of one of the three methyltransferases present in the BGC, *tpcA*, identified it as encoding the emodin *O*-methyltransferase.

The identity of TpcA as the emodin *O*-methyltransferase allows, through comparative bioinformatics and a process of elimination, the assignment of the other methyltransferases in both the trypacidin 21 and geodin 19 BGCs. Firstly, GedA can be assigned as the emodin *O*-methyltransferase due to clear homology with TpcA (72.7% protein identity). This is consistent with the observation by Boruta and Bizukojc^95^ that the sequence predicted mass of GedA (53.6 kDa) but not GedG (36.8 kDa) matches the subunit mass of emodin *O*-methyltransferase originally calculated by Chen *et al.*^19^ The remaining homologous *O*-methyltransferases from the two pathways; GedG and TpcM, can then be assigned the role of methylating the carboxyl group of desmethylsulochrin 16 to give sulochrin 15 (Scheme 13). The identity of GedG and TpcM as catalysing a methyl esterification is consistent with the presence of homologues in the gene clusters of other xanthones with a methyl ester, namely the secalonic acids and neosartorin 38 (See Section 3 and Table 1). Finally, this leaves the unique methyltransferase encoded by the trypacidin BGC; TpcH, as catalysing the methylation of sulochrin 15.

The above studies can now be combined to propose the full pathways from emodin 1 to either geodin 19 or trypacidin 21, with genes and their encoded enzymes assigned to each step (Scheme 13). Both pathways share the common route to 1 (Scheme 2), which is then methylated by emodin *O*-methyltransferase (GedA/TpcA) to give questin 9. As discussed in Section 3.1, reduction of questin to a hydroquinone (GedF/TpcG), followed by dioxygenase catalysed ring cleavage (GedK/TpcI) produces desmethylsulochrin 16. A methyl esterification of 16 by GedG/TpcM then gives sulochrin 15, which is the last common intermediate in the two pathways. Halogenation of sulochrin 15 by GedL give dihydrogeodin^27^ whereas methylation by TpcH gives monomethylsulochrin 73.^28,29^ In both pathways a multicopper oxidase (MCO) GedJ/TpcJ, catalyses the ring closure from the benzophenone precursor to form the grisan structure of the final compound.

Additional grisandienes that arise from the geodin biosynthetic pathway include erdin 20 and bisdechlorogeodin 74. These compounds likely come about through one or more of the pathway enzymes not acting. Bisdechlorogeodin 74 is likely produced due to the MCO GedJ acting on sulochrin 15 without GedL first catalysing the double chlorination seen in the pathway to geodin 19 (Scheme 13). Erdin 20, which was isolated alongside geodin 19 in 1936,^7^ has been proposed to come about through hydrolysis of the ester bond of 19,^96^ but it is also possible that it arises if the carboxyl *O*-methyltransferase GedG does not act.

A remaining question concerning the grisandiene BGCs is the role of the homologous glutathione S-transferases (GSTs) GedE/TpcF. There is a predicted GST encoded in the *mdp* BGC (MdpJ), but no definitive role has been uncovered for MdpJ and furthermore GedE/TpcF show little homology with this protein.

As a final note on grisandienes, it must be mentioned that this structural class contains compounds that do not belong to the emodin family of natural products – providing a clear example of chemical convergent evolution (Box 2).

**Box 2: Griseofulvin as an example of chemical convergent evolution**

**Scheme 14:**
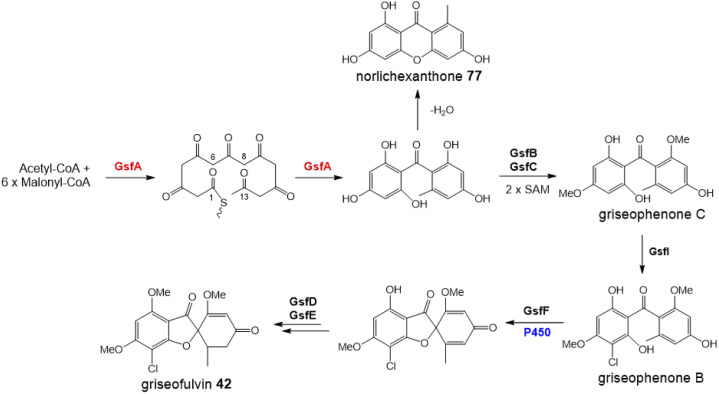
Griseofulvin 42 biosynthesis in *Penicillium aethiopicum*. Adapted from Cacho *et al.*^85^

Structural similarity does not necessarily mean biosynthetic homology, and the griseofulvin 42 pathway of *P. aethiopicum*, which has now been fully elucidated,^85^ is a clear example of this. The griseofulvin PKS, GsfA, is a group V3 nrPKS that catalyses a C1–C6 Claisen condensation and a C8–C13 aldol cyclization to directly produce a benzophenone intermediate, without progressing *via* an anthraquinone intermediate and the action of a ring cleaving dioxygenase (Scheme 14).^85,98^ Expression of GsfA in yeast showed that the common xanthone norlichexanthone 77 is a product of GsfA in *P. aethiopicum* and arises *via* spontaneous dehydration of the benzophenone.^85^ Bis-*O*-methylation of the benzophenone intermediate in the griseofulvin pathway by GsfB and GsfC is thought to hinder xanthone formation.

Another key difference between the emodin-related grisandienes and griseofulvin 42 is the key ring closure to form the spiran core. Rather than a multi-copper oxidase, as is seen in the geodin 19, trypacidin 21 and pestheic acid 22 pathways, in griseofulvin 42 biosynthesis a P450 called GsfF performs the oxidative coupling between the orcinol and the phloroglucinol rings to yield the grisan structure. Again, this demonstrates how these structurally homologous natural products can come about through biosynthetically diverse means.

#### 4.3.2 Diphenyl ethers

A structural class of compounds closely related to the grisandienes are the diphenyl ethers. Diphenyl ethers that likely arise from the geodin 19 pathway include asterric acid **24**, demethylasterric acid 75 and geodin hydrate 76 (Scheme 13). Asterric acid 24 was first reported in 1960,^97^ and more recently the aforementioned diphenyl ethers have all been isolated alongside questin 9, desmethylsulochrin 16, sulochrin 15 and geodin 19 during a bioreactor-based analysis of *A. terreus* strain ATCC 20542.^96^ Asterric acid 24 has been confirmed as being derived from the geodin BGC as disruption of the PKS – later identified as the ACAS GedC – abolished asterric acid 24 production.^41^24, 75 and 76 are thought to be the products of the retro-aldol cleavage of bisdechlorogeodin 74, erdin 20 and geodin 19 respectively (Scheme 13). As mentioned previously, 74 is likely to come about due to the MCO of the geodin pathway acting on sulochrin 15 without the action of the halogenase GedL. Consistent with this is the recent isolation of both sulochrin 15 and asterric acid 24, but not geodin 19, from cultures of *Scytalidium album*, and the identification of a BGC that is identical to the geodin cluster except that it is missing a homologue to the halogenase GedL (unpublished data).

At the same time that the geodin biosynthetic gene cluster was being investigated by Nielsen and colleagues,^99^ the BGC of the diphenyl ether pestheic acid 22 was being investigated by Liu and colleagues.^30^ Pestheic acid 22, originally named dihydromaldoxin, is a plant growth regulator^100^ that was first isolated in 1996 from an unidentified *Xylaria* species^101^ and later isolated from *Pestalotiopsis theae*^100^ and *Pestalotiopsis fici.*^102^ To identify the pestheic acid BGC, the genome of *P. fici* CGMCC3.15140 was sequenced and a putative gene cluster was identified, which contained a typical group V1 nrPKS. Disruption of this PKS, named *ptaA*, abolished the production of pestheic acid 22, thus confirming the identity of the *pta* cluster.^30^ A bioinformatic analysis of the BGC identified a range of predicted proteins likely to be involved in pestheic acid 22 biosynthesis, including the DHGO (dihydrogeodin oxidase)-like enzymes; PtaE and PtaK. Both enzymes were disrupted, and in the case of PtaE, this led to a loss of pestheic acid 22 and its non-chlorinated analogue RES-1412-1, and an accumulation of isosulochrin 78 and chloroisosulochrin 79.^30^

Disruption of a putative flavin-dependant halogenase PtaM from the *pta* cluster, led to the accumulation of the non-chlorinated isosulochrin 78 as well as RES-1214-1 23 but a loss of the chlorinated compounds chloroisosulochrin 79 and pestheic acid 22. PtaM was then investigated through *in vitro* assays and was shown to convert isosulochrin 78 to chloroisosulochrin 79 but could not convert RES-1214-1 23 to pestheic acid 22, demonstrating strict substrate specificity and showing that halogenation must occur prior to diphenyl ether formation. It was also shown that PtaM can catalyse bromination of isosulochrin 78 to give a brominated analogue of chloroisosulochrin.^30^

Comparisons with the now known geodin and trypacidin BGCs allows the methyltransferases in the pestheic acid cluster to be putatively assigned. PtaI is unique to the pestheic acid cluster, whereas PtaH is homologous to gedG and tpcM, as well as *O*-methyltransferases in other pathways that involve a carboxyl *O*-methylation (see Section 3.3). Therefore, PtaI can be confidently assigned as catalysing the methylation of emodin 1 to physcion 10, and PtaH can been assigned as catalysing the conversion of desmethylisosulochrin 80 to isosulochrin 78 (Scheme 13).

#### 4.3.3 Spiroketals

**Scheme 15:**
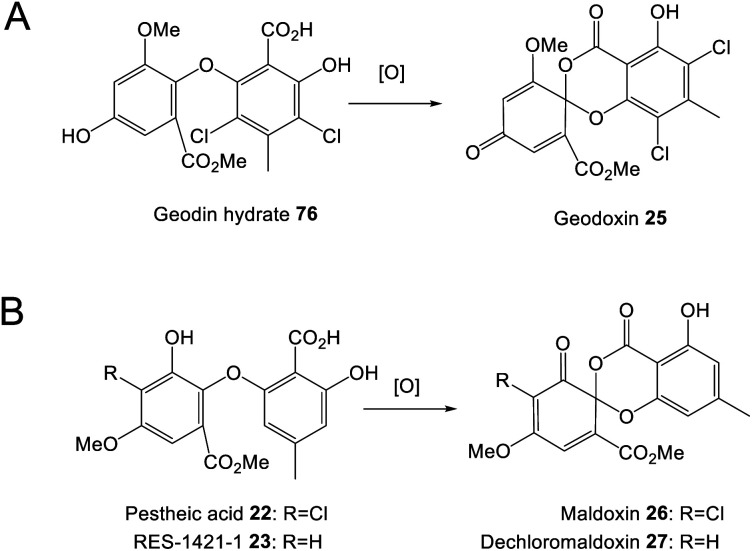
Diphenyl ethers can undergo oxidative coupling to produce spiroketals with an unusual 1,3-benzodioxin-4-one core such as geodoxin 25 (A) maldoxin 26. and dechloromaldoxin 27 (B).

A final class of compounds worthy of mention are the diphenyl ether derived spiroketals. Geodoxin 25 was isolated from *Aspergillus terreus* as early as 1959,^93^ and quickly shown to come about through an oxidative coupling of geodin hydrate 76^103,104^ (Scheme 15A). Maldoxin 26, which was isolated from a *Xylaria* species alongside pestheic acid 22 (a.k.a. dihydromaldoxin)^101^ is likely to come about through an analogous oxidative coupling (Scheme 15B), with dechloromaldoxin 27 – isolated from *P. fici* in 2013^105^ – being the probable product of oxidative coupling of RES-1412-1 23 (Scheme 15B).

**Scheme 16:**
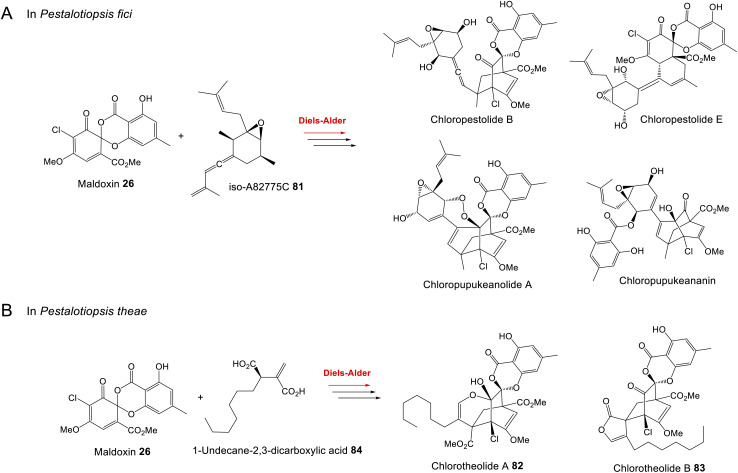
In *P. fici* (A) and *P. theae* (B) maldoxin is a precursor, along with either iso-A82775C 81 or 1-undecen-2,3-dicarboxylic acid 84 respectively, for unusual Diels–Alder cascades that produce a wide range of compounds. In *P fici*, these include the chloropupakeananes and chloropestiolides (a selection of which are shown), and in *P. theae* compounds isolated so far include chlorotheolides A 82 and B 83.

Although the relationship between maldoxin 26, dechloromaldoxin 27 and the pestheic acid cluster has not been absolutely proven, it is supported by the presence of complex compounds named chloropupukeananes and chloropestolides that are produced by *Pestalotiopsis fici*.^102,106^ Chloropupukeananes and chloropestolides either contain, or are derived from, a 1,3-benzodioxin-4-one core structure and are thought to be derived from maldoxin 26 and a prenylated compound called iso-A82775C 81 through Diels–Alder reaction cascades^105,107^ (Scheme 16A). Disruption of the pestheic acid nrPKS (*ptaA*) abolished production of the chloropupukeananes, confirming that pestheic acid 22 is one of the precursors of chloropupukeananes, with maldoxin 26 being a presumed intermediate. The inability to isolate maldoxin 26 itself from *P. fici* was speculated as being due to the high reactivity with iso- A82775C 81. It was proposed by Xu *et al.*, that the dihydrogeodin oxidase-like multicopper enzyme PtaK, which has been ruled out as being necessary for pestheic acid biosynthesis, may actually be involved in the oxidative coupling to produce maldoxin 26.^30^

In *P. theae*, maldoxin 26 was isolated alongside spiroketals called chlorotheolides A 82 and B 83 (Scheme 16B).^108^ These compounds are thought to be products of Diels–Alder reactions between maldoxin 26 and another compound named 1-undecen-2,3-dicarboxylic acid 84.^108^

## 5 Conclusions

After nearly a century of chemical and biosynthetic studies, and a decade of genomics driven research, a detailed understanding is now emerging regarding the biosynthesis of this important and prolific family of fungal natural products. Feeding studies, gene editing, heterologous expression and *in vitro* work has combined to illuminate the biosynthetic pathways to the key anthraquinone intermediates, including endocrocin, emodin and chrysophanol; with nearly all of the enzymatic steps well understood.

The recent demonstrations that hydroquinones rather than anthraquinones are required as substrates for various enzymatic reactions has highlighted the vital role of anthraquinone reductases, and has explained their presence in not only pathways where deoxygenation of emodin is required, but also pathways such as the grisandienes, where reduction of the anthraquinone is required prior to ring cleavage.

We now also know the key transformations required to generate the different core structures, including the reductase and dioxygenase-catalysed ring cleavage of anthraquinones, the multi-copper oxidases involved in spiran formation in the pathways to grisandienes and diphenyl ethers, and the recently uncovered snoAL-like proteins involved in the formation of xanthones. The many dimeric compounds in this family appear to be exclusively produced by the oxidative action of P450 dimerase enzymes, and the finer details regarding pathway specificity and stereochemical control are rapidly being uncovered.

An understanding of the ‘core’ enzymes that are common within structurally and biosynthetically related pathways, paves the way for genome-mining approaches to uncover further diversity. In such a bioactive family of natural products, this could have untold applications. The elucidation of pathway specific tailoring steps also provides us with great enzymatic potential, which could have a host of applications, for example in combinatorial biosynthesis, or for use as biocatalysts.

## 6 Author contributions

KdMS devised the concept of the review, conducted the comparative bioinformatics and took the lead on writing the manuscript. TS contributed to the writing and provided continual feedback, as well as contributing to pathway proposals and providing mechanistic expertise.

## 7 Conflicts of interest

There are no conflicts to declare.

## Supplementary Material
