# Correction: The ‘emodin family’ of fungal natural products–amalgamating a century of research with recent genomics-based advances

**DOI:** 10.1039/d4np90035a

**Published:** 2024-08-09

**Authors:** Kate M. J. de Mattos-Shipley, Thomas J. Simpson

**Affiliations:** a School of Biological Sciences, University of Bristol 24 Tyndall Avenue Bristol BS8 1TQ UK kate_dms@hotmail.com; b School of Chemistry, University of Bristol Cantock's Close Bristol BS8 1TS UK

## Abstract

Correction for ‘The ‘emodin family’ of fungal natural products–amalgamating a century of research with recent genomics-based advances’ by Kate M. J. de Mattos-Shipley *et al.*, *Nat. Prod. Rep.*, 2023, **40**, 174–201, https://doi.org/10.1039/D2NP00040G.

The authors regret that the MRC funder grant number MR/N029909/1 was omitted from the original article. The grant number is included herein to provide the funding information for this article.

The Royal Society of Chemistry apologises for these errors and any consequent inconvenience to authors and readers.

